# Supplementary material for: Isolation and Structural Identification of New Diol Esters of Okadaic Acid and Dinophysistoxin-1 from the Cultured Prorocentrum lima
Source: Toxins (Basel). 2025 Jan 7;17(1):28. doi: 10.3390/toxins17010028 (PMC11768745; doi:10.3390/toxins17010028)
Supplement: Supplementary file 1 [file toxins-17-00028-s001.zip › toxins-3331974-supplementary.pdf]

Supplementary Materials

Isolation and Structural Identification of new Diol Esters of Okadaic Acid and Dinophysistoxin-1 from the Cultured *Prorocentrum lima*

Contents

Table S1. (A) OA diol and (B) DTX-1 diol derivates previously isolated from *Prorocentrum / Dinophysis* species.······························ 3

Table S2. Spectral Data for Dinophysistoxin-1 (DTX-1) and DTX-1 part of compound **1** in CD_3OD.--------5

Figure S1. HRESIMS data for compound **1**. --6

Figure S2. (A) ^1H NMR and (B)^{13}C NMR spectra of compound **1** measured at 500 MHz(^1H). ---------------7

Figure S3. COSY spectrum of compound **1** measured at 500 MHz(^1H). --8

Figure S4. HSQC spectrum of compound **1** measured at 500 MHz(^1H). --9

Figure S5. HMBC spectrum of compound **1** measured at 500 MHz(^1H). ---------------------------------------10

Figure S6. DP4+ probability analysis for the diol moiety of compound **1**. -------------------------------------11

Figure S7. HRESIMS data of compound **2**. --12

Figure S8. (A) ^1H NMR and (B)^{13}C NMR spectra of compound **2** measured at 900 MHz(^1H). ---------------13

Figure S9. COSY spectrum of compound **2** measured at 900 MHz(^1H). --14

Figure S10. HSQC spectrum of compound **2** measured at 900 MHz(^1H). ------------------------------------15

Figure S11. HMBC spectrum of compound **2** measured at 900 MHz(^1H). ------------------------------------16

Figure S12. ROESY spectrum of compound **2** measured at 900 MHz(^1H). ------------------------------------17

Figure S13. HRESIMS data of compound **3**. ---18

Figure S14. (A) ^1H NMR and (B)^{13}C NMR spectra of compound **3** measured at 500 MHz(^1H). -------------19

Figure S15. COSY spectrum of compound **3** measured at 500 MHz(^1H). ---------------------------------------20

Figure S16. HSQC spectrum of compound **3** measured at 500 MHz(^1H). ------------------------------------21

Figure S17. HMBC spectrum of compound **3** measured at 500 MHz(^1H). ------------------------------------22

Figure S18. DP4+ probability analysis for the diol moiety of compound **3**. -----------------------------------23

Figure S19. HRESIMS data of compound **4**. ---24

Figure S20. (A) ^1H NMR and (B)^{13}C NMR spectra of compound **4** measured at 900 MHz(^1H). -------------25

Figure S21. COSY spectrum of compound **4** measured at 900 MHz(^1H). ---------------------------------------26

Figure S22. HSQC spectrum of compound **4** measured at 900 MHz(^1H). ------------------------------------27

Figure S23. HMBC spectrum of compound **4** measured at 900 MHz(^1H). ------------------------------------28

Figure S24. ROESY spectrum of compound **4** measured at 900 MHz(^1H). ------------------------------------29

Figure S25. Apoptosis of (A) Neuro2a, (B) HCT116, and (C) HepG2 cells in the presence of compounds **1**–**4** or okadaic acid as determined using the Annexin-V/PI double staining assay. ------------ 30

Table S1. (A) OA diol and (B) DTX-1 diol derivates previously isolated from *Prorocentrum / Dinophysis* species.

(A)

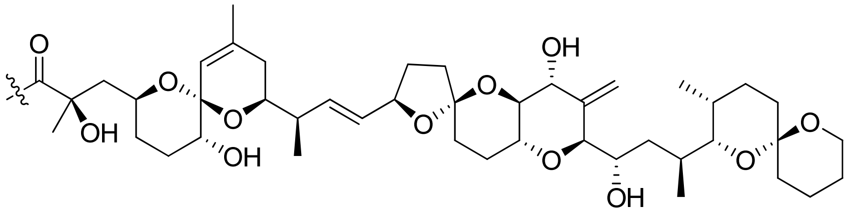

OA-diols	structure	molecular formula $[M+NH_4]^+$	Ref.
D4	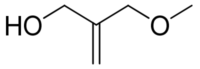	$C_{48}H_{74}O_{14}$ 892.5422	23
D6	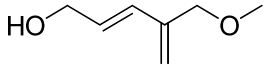	$C_{50}H_{76}O_{14}$ 918.5597	8
D6	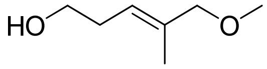	$C_{50}H_{78}O_{14}$ 920.5735	15
D7	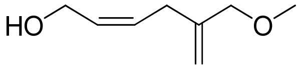	$C_{51}H_{78}O_{14}$ 932.5732	24
D7	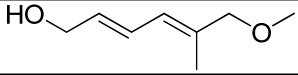	$C_{51}H_{78}O_{14}$ 932.5732	6
D8	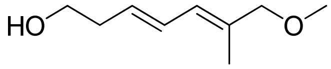	$C_{52}H_{80}O_{14}$ 946.5892	6
D8	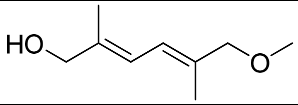	$C_{53}H_{82}O_{14}$ 960.6048	17
D8	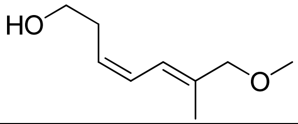	$C_{52}H_{80}O_{14}$ 946.5892	25
D8	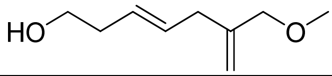	$C_{52}H_{80}O_{14}$ 946.5892	This study
D9	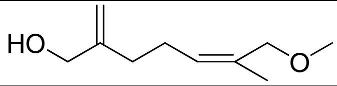	$C_{53}H_{82}O_{14}$ 960.6048	24
D9	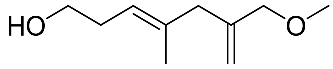	$C_{53}H_{82}O_{14}$ 960.6048	6
D9	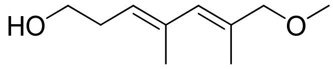	$C_{53}H_{82}O_{14}$ 960.6048	9
T9	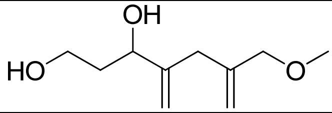	$C_{53}H_{82}O_{15}$ 970.5997	8
D9	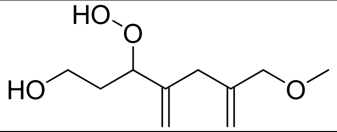	$C_{53}H_{82}O_{16}$ 992.5947	8

D9	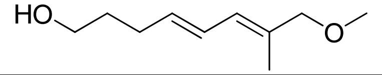	$C_{53}H_{82}O_{14}$ 960.6048	16
D9	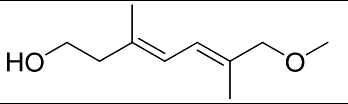	$C_{53}H_{82}O_{14}$ 960.6048	16
D9	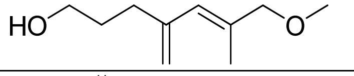	$C_{53}H_{82}O_{14}$ 960.6048	This study
D10	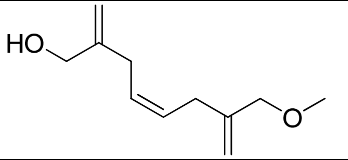	$C_{54}H_{82}O_{14}$ 972.6048	8
D10	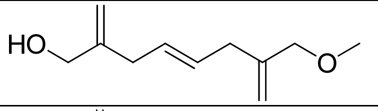	$C_{54}H_{82}O_{14}$ 972.6048	11
D10	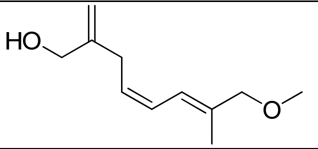	$C_{54}H_{84}O_{14}$ 972.6048	15
D10	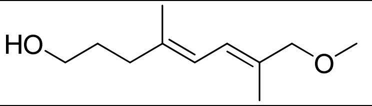	$C_{54}H_{82}O_{14}$ 970.6205	16
D11	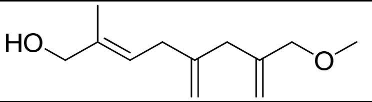	$C_{55}H_{84}O_{14}$ 986.6205	This study

(B)

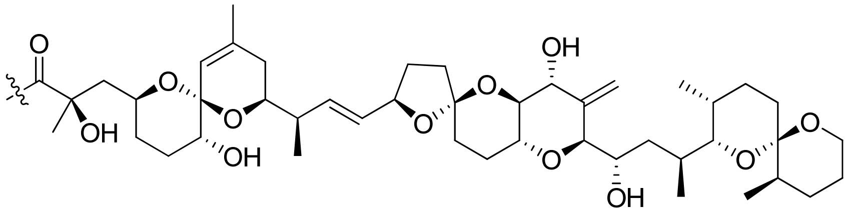

DTX-1-diols	structure	molecular formula $[M+NH_4]^+$	Ref.
D6	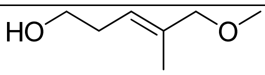	$C_{51}H_{80}O_{14}$ 934.5892	15
D9	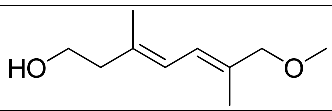	$C_{54}H_{84}O_{14}$ 974.6205	10

Table S2. Spectral Data for Dinophysistoxin-1 (DTX-1) and DTX-1 part of compound **1** in CD_3OD (500 MHz, ^1H).

	δ_H (**1**)	δ_C (**1**)	δ_C (DTX-1)
1		176.2.0, C	182.7
2		75.5, C	76.5
3	1.89 (m)	46.5, CH_2	46.6
4	3.97 (t, 2.5)	68.1, CH	69.0
5	1.38 (m); 1.74 (m)	33.4, CH_2	33.4
6	1.65 (m); 1.96 (m)	28.0, CH_2	28.2
7	3.35 (m)	73.0, CH	73.4
8		97.6, C	97.6
9	5.23 (br s)	123.4, CH	123.7
10		139.6, C	139.4
11	1.83 (m); 1.91 (m)	34.0, CH_2	34.0
12	3.64 (m)	72.3, CH	71.8
13	2.33, m	42.9, CH	43.3
14	5.77 (dd, 15.2, 8.1)	136.5, CH	137.6
15	5.54 (dd, 15.2, 7.6)	132.3, CH	131.9
16	4.56 (m)	80.4, CH	80.6
17	1.62 (m); 2.20 (m)	31.6, CH_2	31.5
18	1.86 (m); 2.00 (m)	38.0, CH_2	38.1
19		107.1, C	107.0
20	1.87 (m)	34.1, CH_2	34.1
21	1.76 (m); 1.89 (m)	27.7, CH_2	27.7
22	3.64 (m)	71.2, CH	71.3
23	3.36 (t, 9.8)	78.3, CH_2	78.2
24	4.10, m	71.7, CH	72.1
25		147.1, C	147.1
26	3.93 (d, 9.1)	86.5, CH	86.4
27	4.10 (m)	66.1, CH	66.1
28	0.94 (m); 1.34 (m)	36.8, CH_2	36.8
29	1.89 (m)	32.4, CH	32.4
30	3.24 (dd, 10.2, 2.0)	76.5, CH	76.5
31	1.79 (m)	28.7, CH	28.7
32	1.40 (m); 1.99 (m)	27.5, CH_2	27.5
33	1.12 (m); 1.95 (m)	26.9, CH_2	26.9
34		99.4, C	99.3
35	1.52 (m)	40.4, CH_2	40.4
36	1.45 (m); 1.63 (m)	28.6, CH_2	28.6
37	1.51 (m); 1.63 (m)	27.4, CH_2	27.5
38	3.48 (m); 3.64 (m)	60.9, CH_2	60.9
39	1.44 (s)	25.7, CH_3	27.9
40	1.73 (s)	23.1, CH_3	23.2
41	1.03 (d, 6.9)	16.5, CH_3	16.7
42	5.03 (br s); 5.32 (br s)	112.4, CH_2	112.3
43	1.03 (d, 6.4)	16.5, CH_3	16.5
44	0.92 (d, 7.1)	11.1, CH_3	11.1
45	0.93 (d, 6.6)	17.2, CH_3	17.2

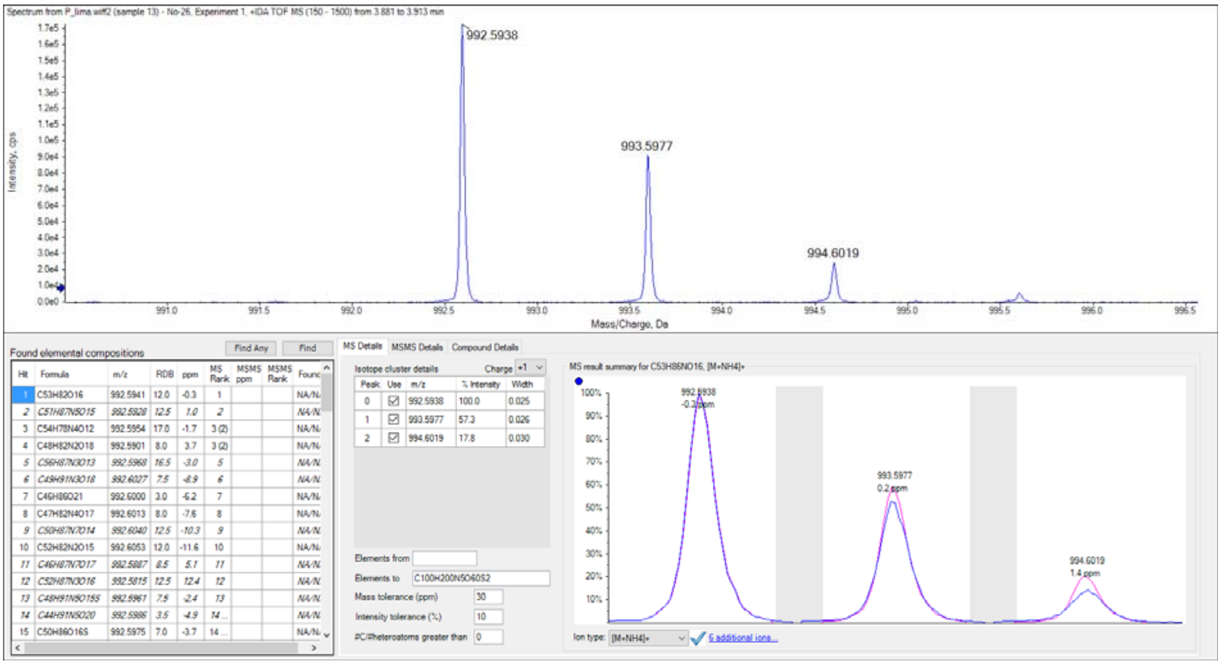

Figure S1. HRESIMS data for compound **1**.

(A)

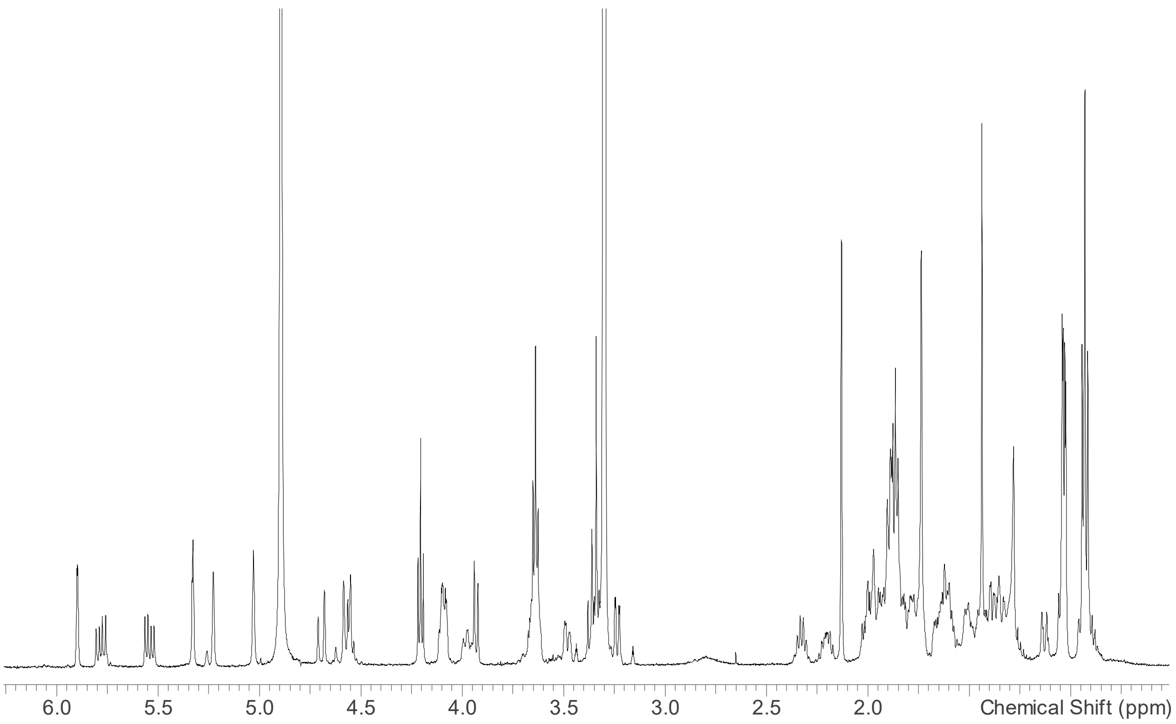

(B)

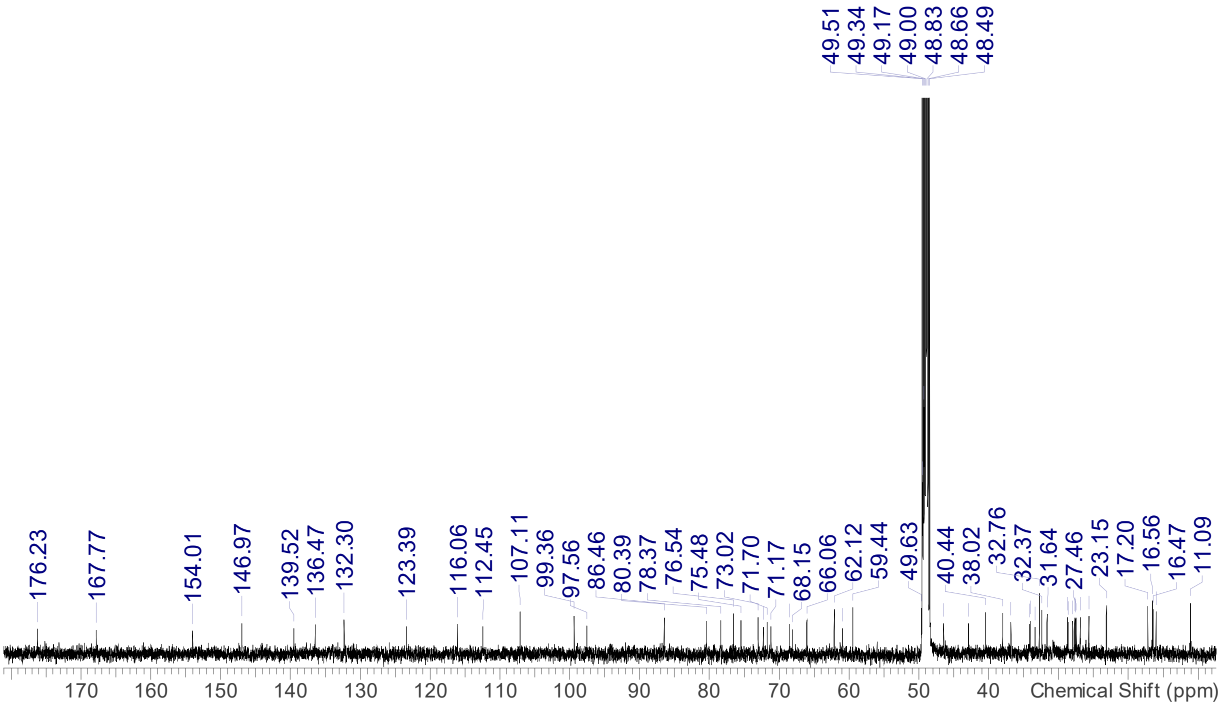

Figure S2. (A) ^{1}H NMR and (B)^{13}C NMR spectra of compound **1** measured at 500 MHz(^{1}H).

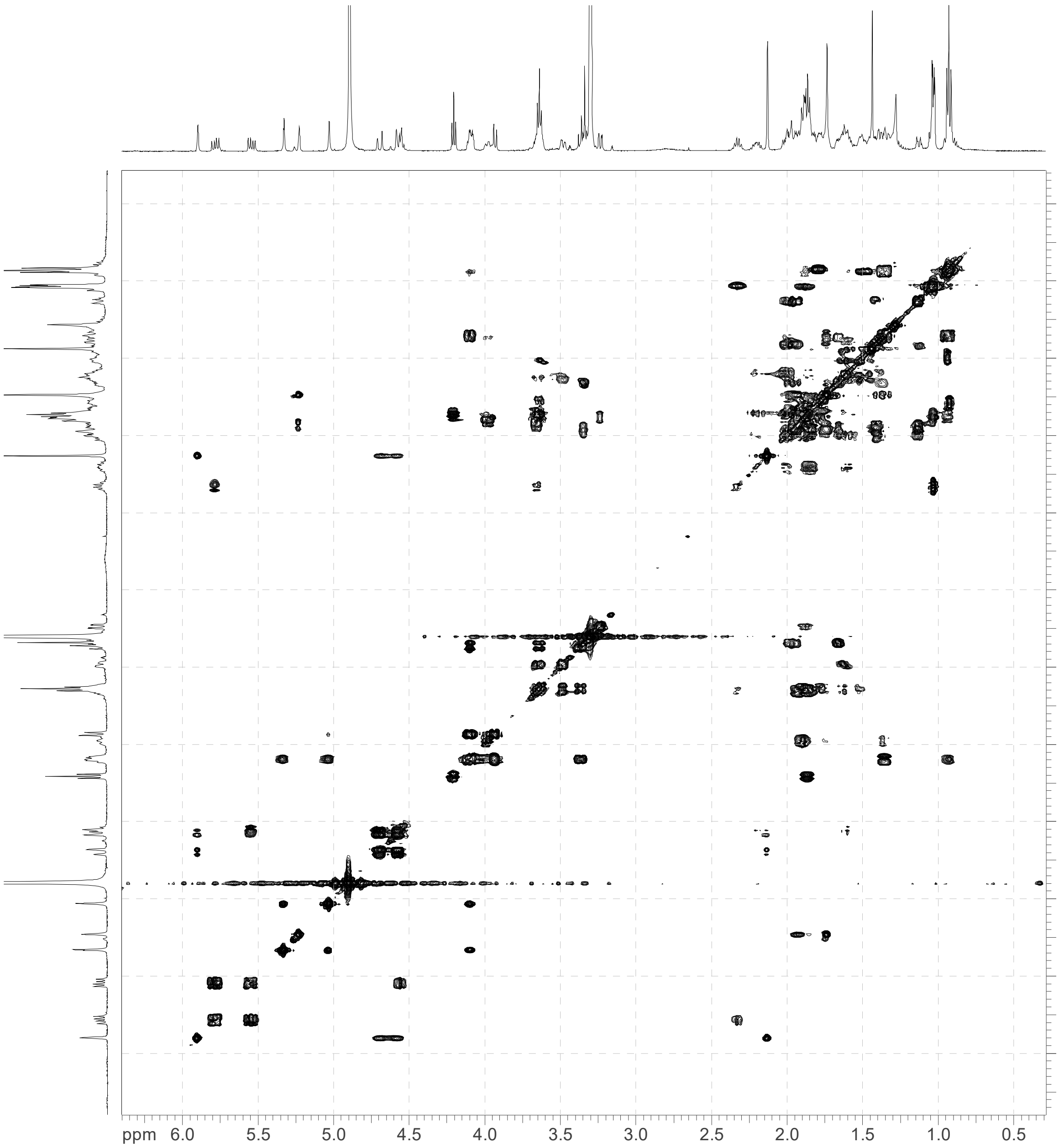

Figure S3. COSY spectrum of compound **1** measured at 500 MHz(^{1}H).

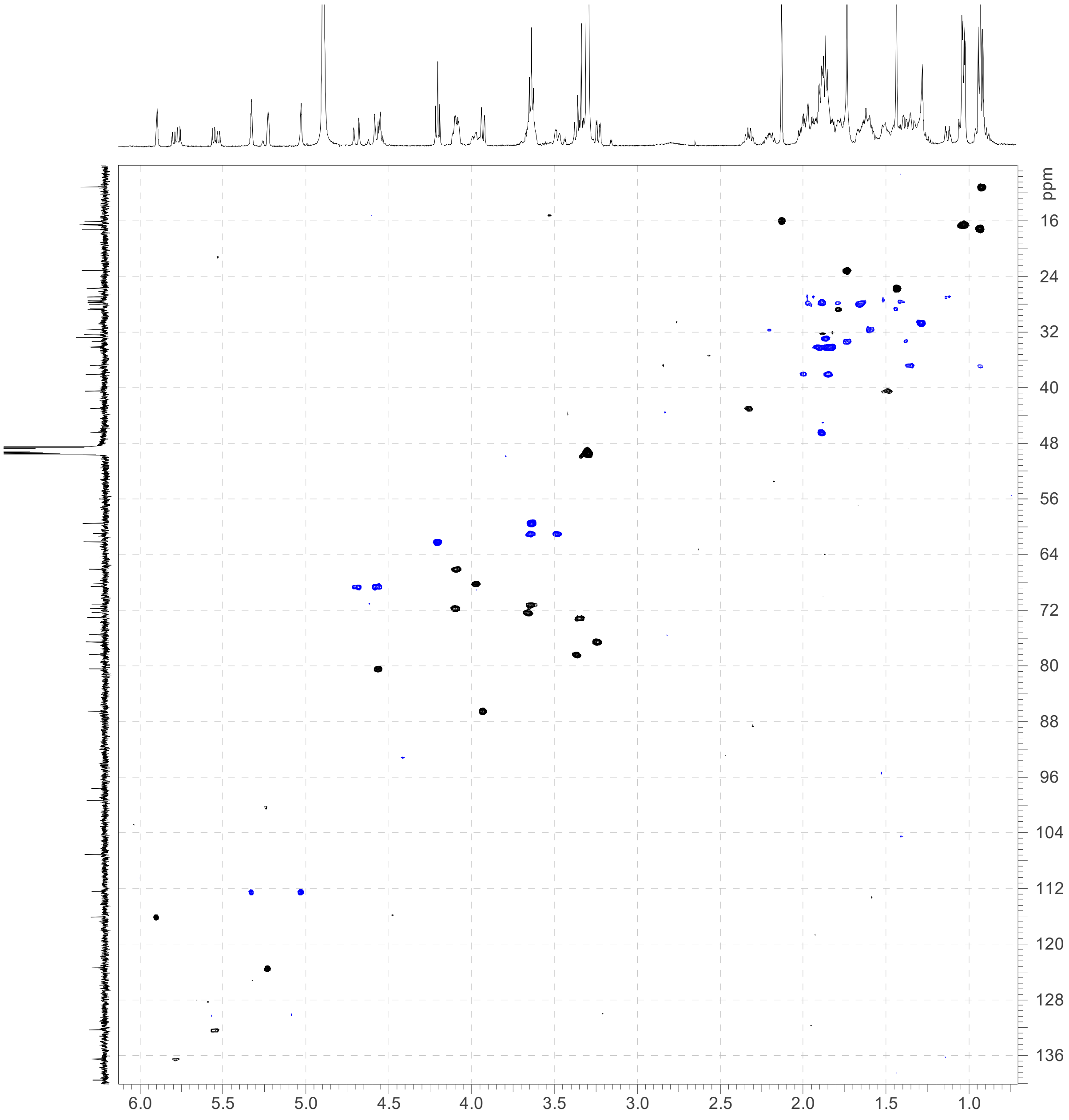

Figure S4. HSQC spectrum of compound **1** measured at 500 MHz(^{1}H).

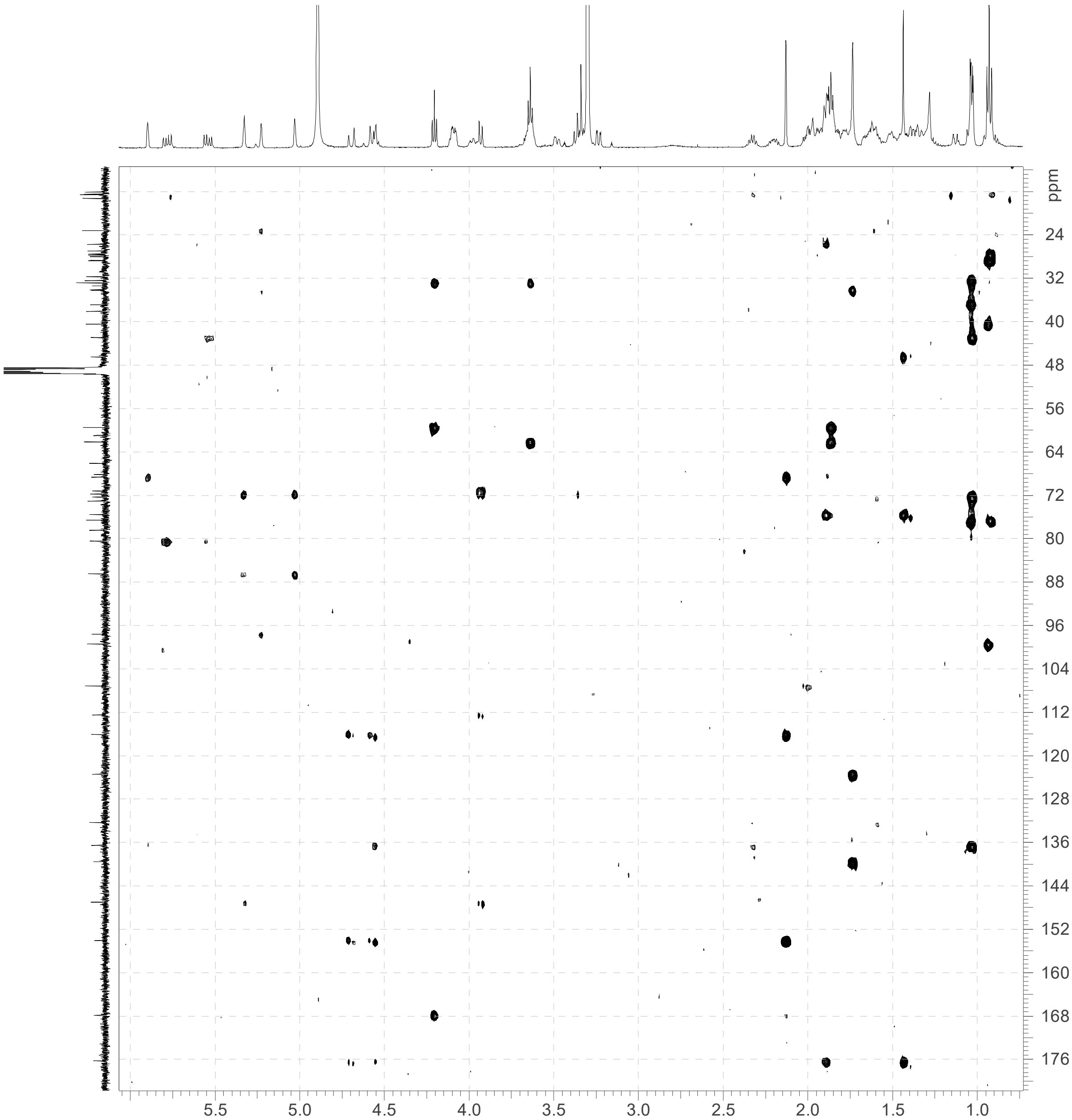

Figure S5. HMBC spectrum of compound **1** measured at 500 MHz(^{1}H).

	A	B	C	D	E	F	G	H
1	Functional		Solvent?		Basis Set		Type of Data	
2	mPW1PW91		PCM		6-311G(d,p)		Shielding Tensors	
3								
4			Isomer 1	Isomer 2	Isomer 3	Isomer 4	Isomer 5	Isomer 6
5	sDP4+ (H data)		41.00%	59.00%	-	-	-	-
6	sDP4+ (C data)		100.00%	0.00%	-	-	-	-
7	sDP4+ (all data)		100.00%	0.00%	-	-	-	-
8	uDP4+ (H data)		98.96%	1.04%	-	-	-	-
9	uDP4+ (C data)		99.85%	0.15%	-	-	-	-
10	uDP4+ (all data)		100.00%	0.00%	-	-	-	-
11	DP4+ (H data)		98.51%	1.49%	-	-	-	-
12	DP4+ (C data)		100.00%	0.00%	-	-	-	-
13	DP4+ (all data)		100.00%	0.00%	-	-	-	-

Isomer 1 = (*E*)-form diol

Isomer 2 = (*Z*) form diol

Figure S6. DP4+ probability analysis for the diol moiety of compound **1**.

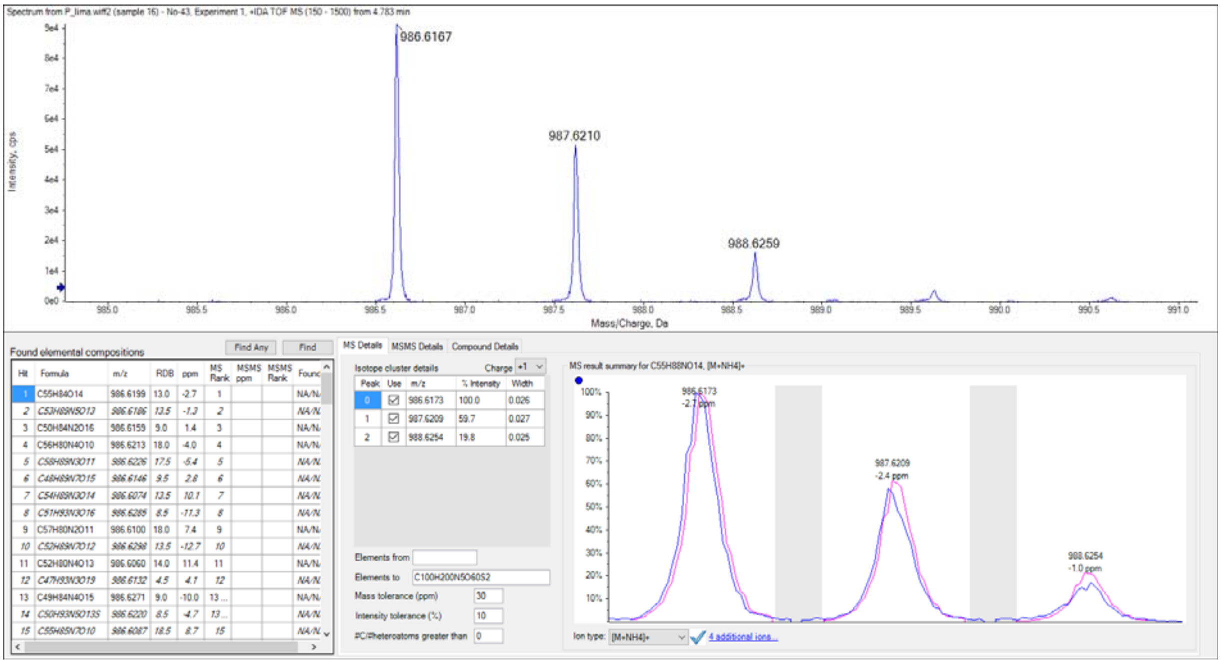

Figure S7. HRESIMS data of compound **2**.

(A)

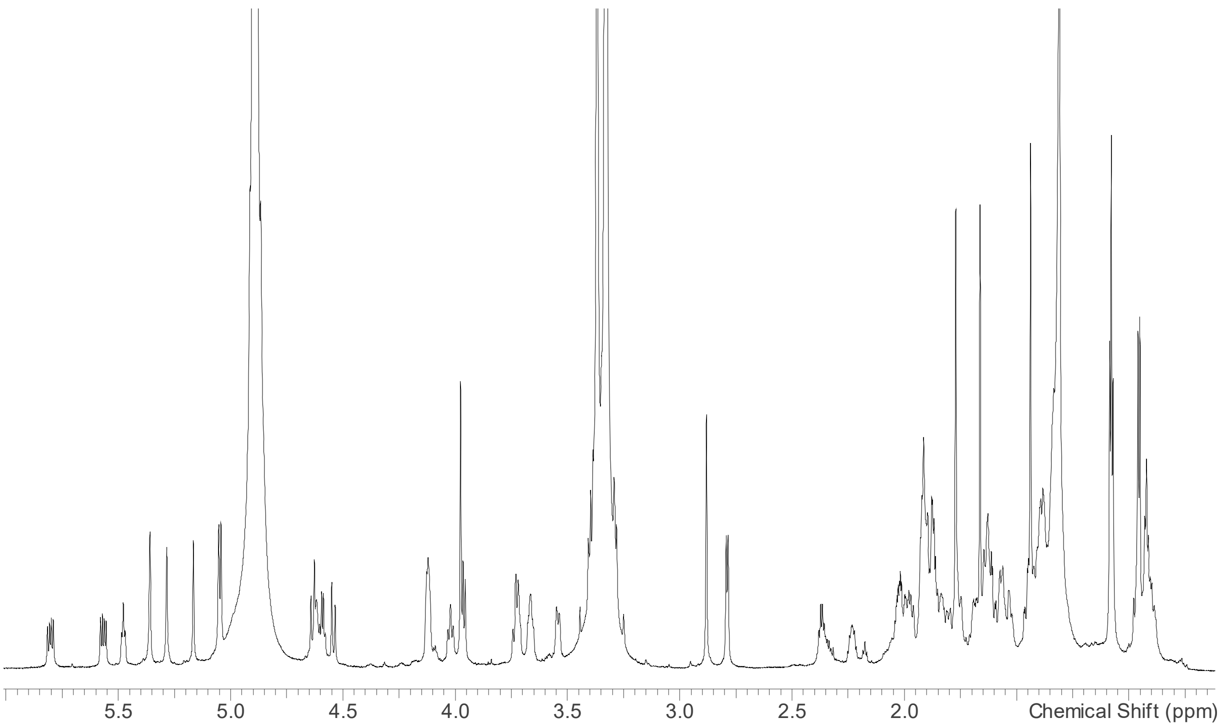

(B)

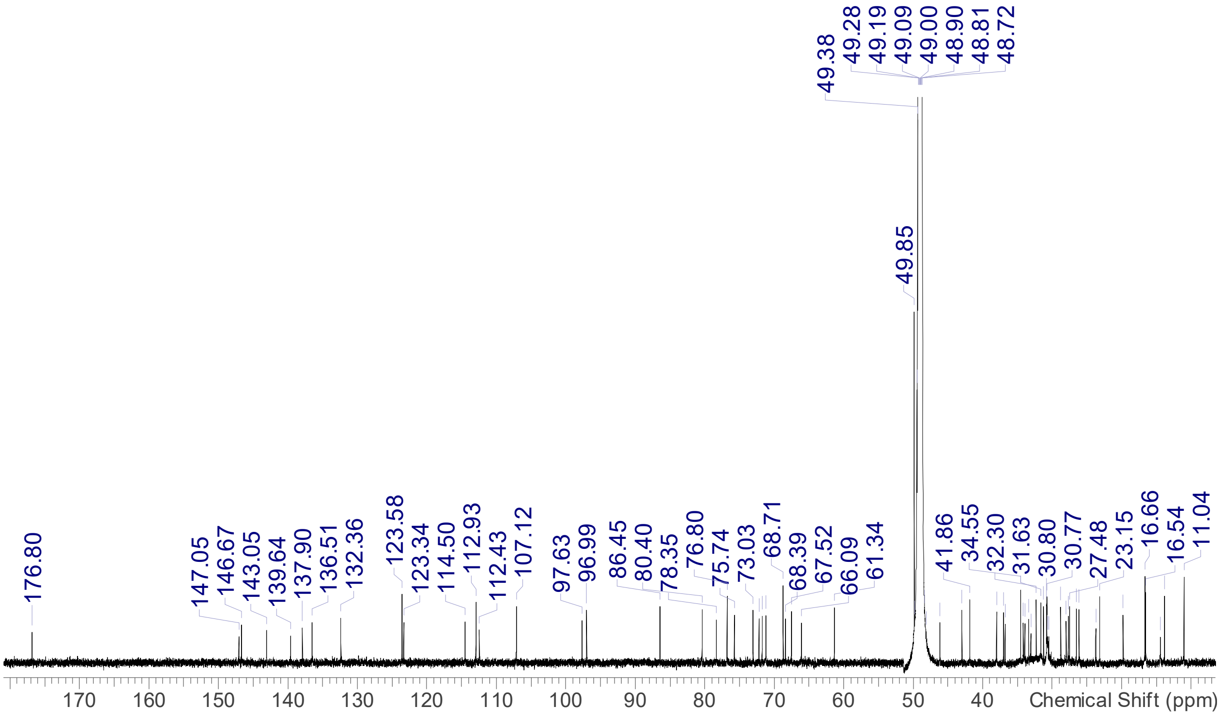

Figure S8. (A) ^{1}H NMR and (B)^{13}C NMR spectra of compound **2** measured at 900 MHz(^{1}H).

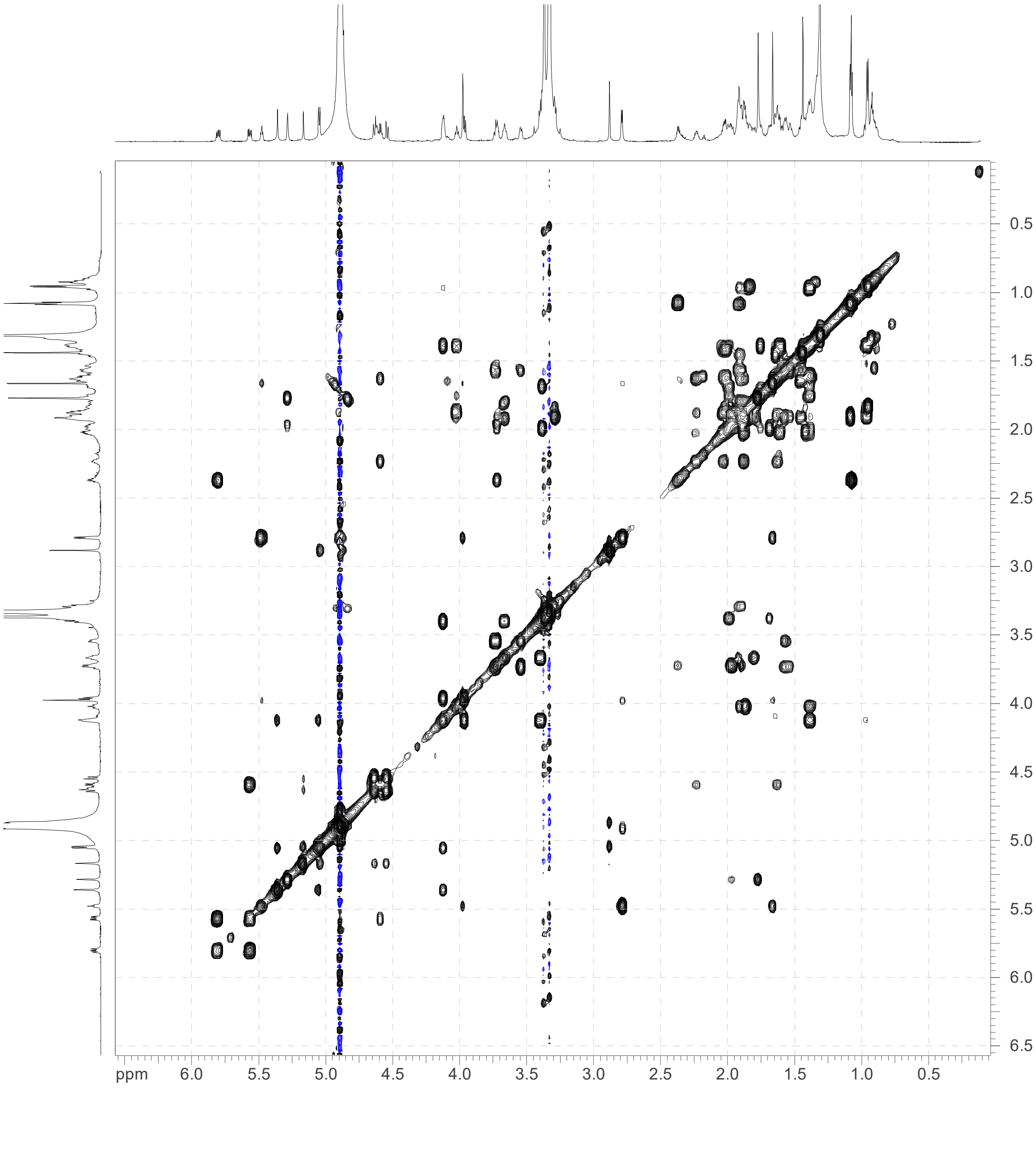

Figure S9. COSY spectrum of compound **2** measured at 900 MHz(^{1}H).

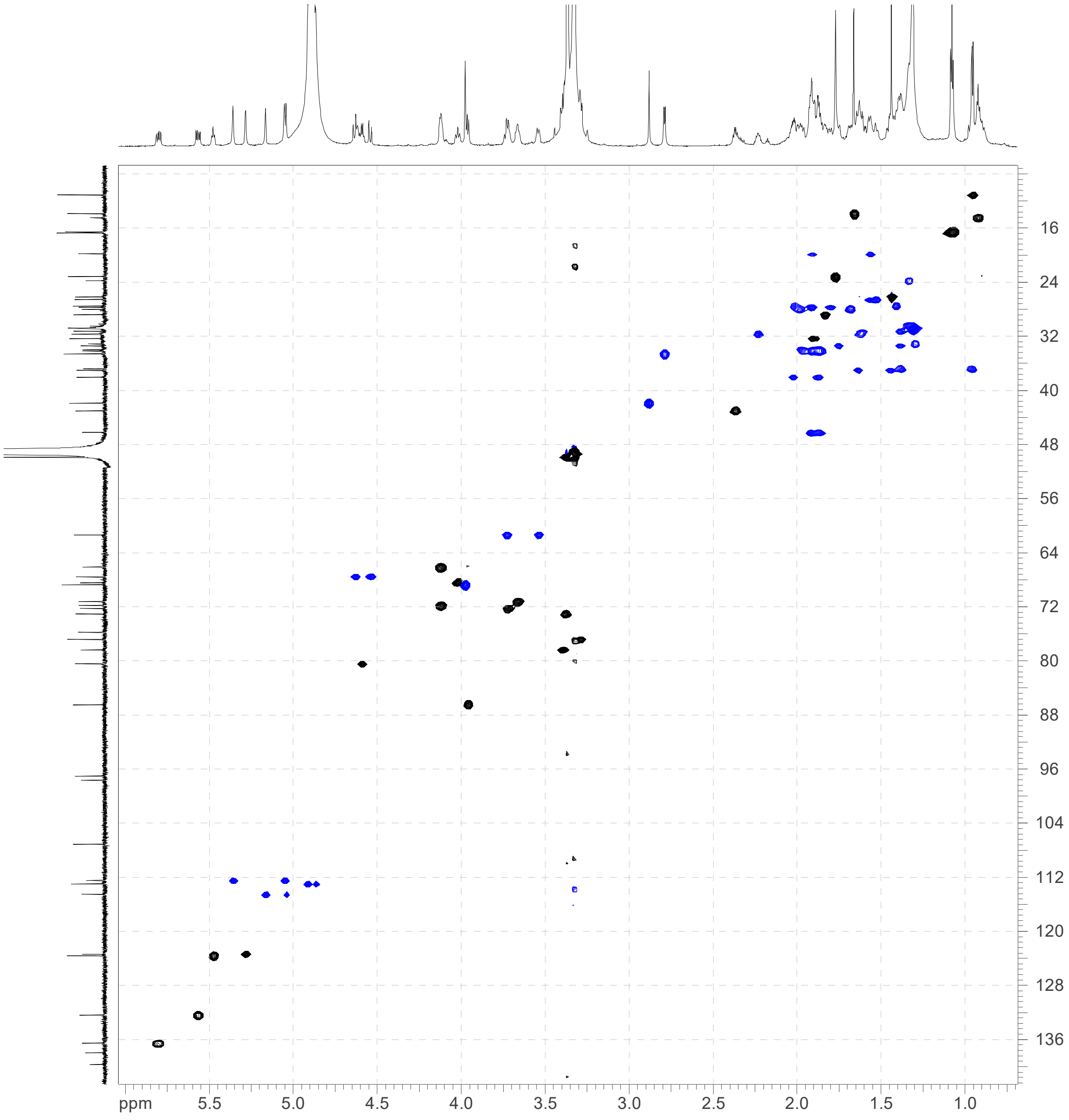

Figure S10. HSQC spectrum of compound **2** measured at 900 MHz(^{1}H).

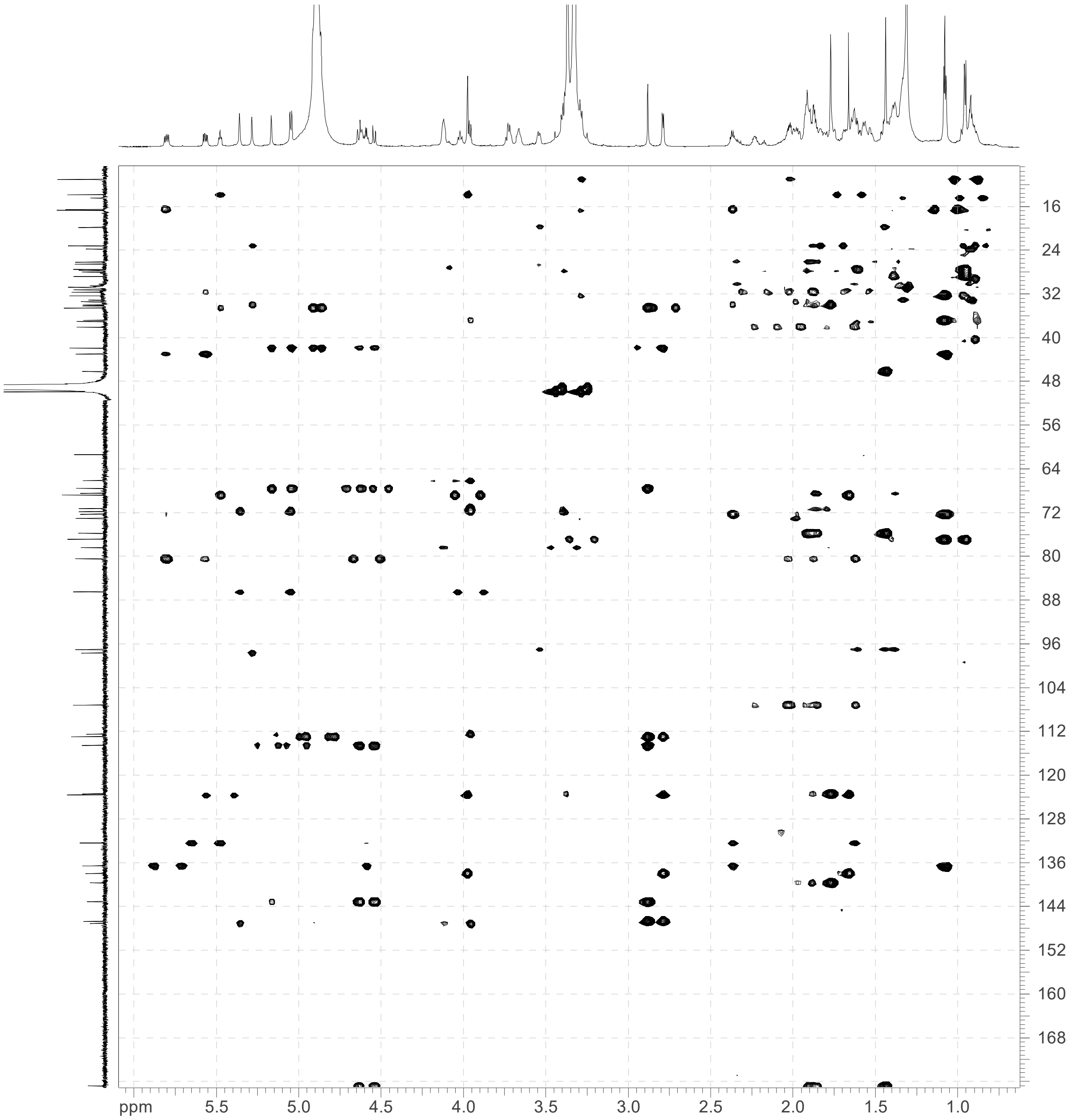

Figure S11. HMBC spectrum of compound **2** measured at 900 MHz(^{1}H).

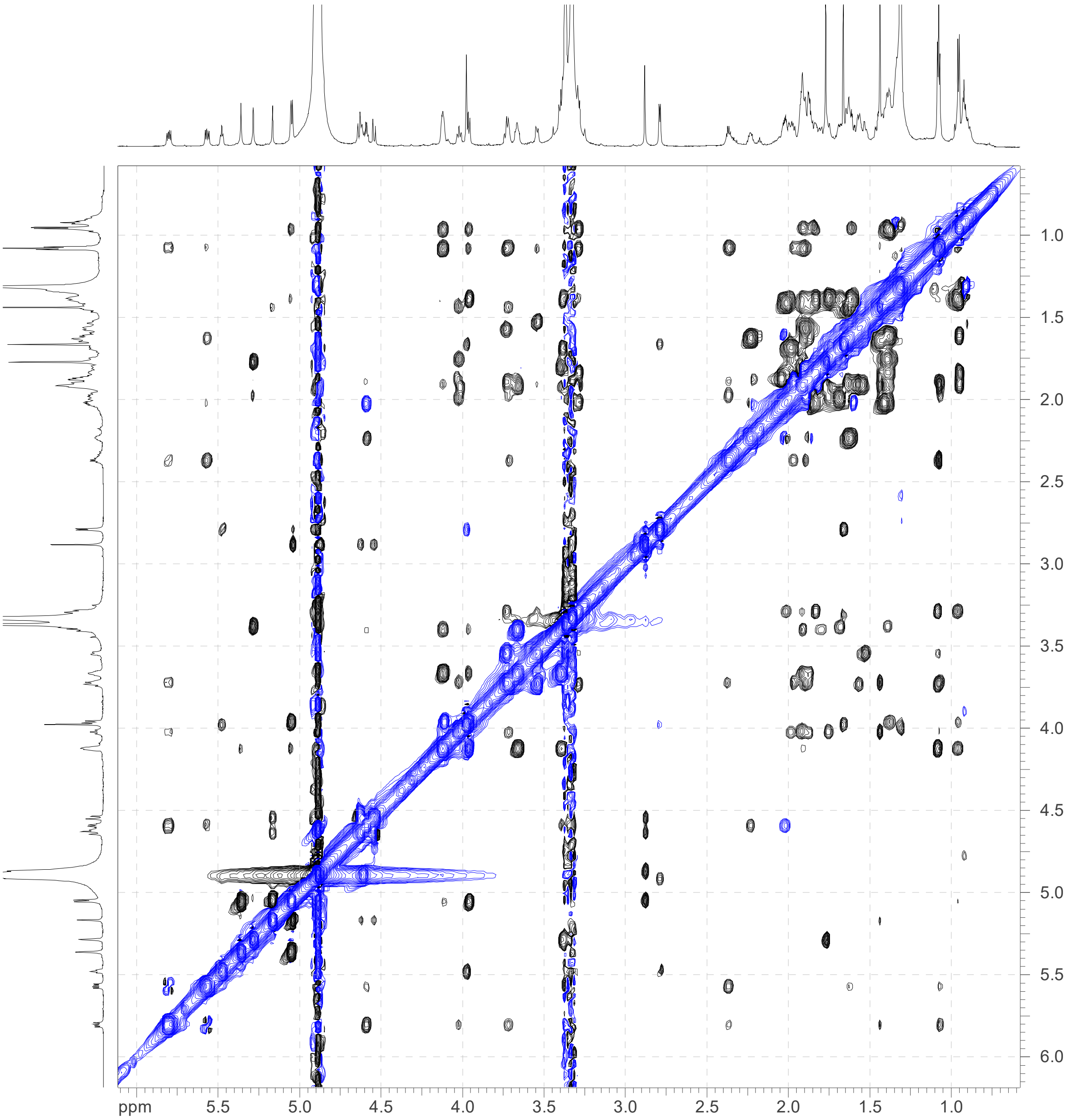

Figure S12. ROESY spectrum of compound **2** measured at 900 MHz(^{1}H).

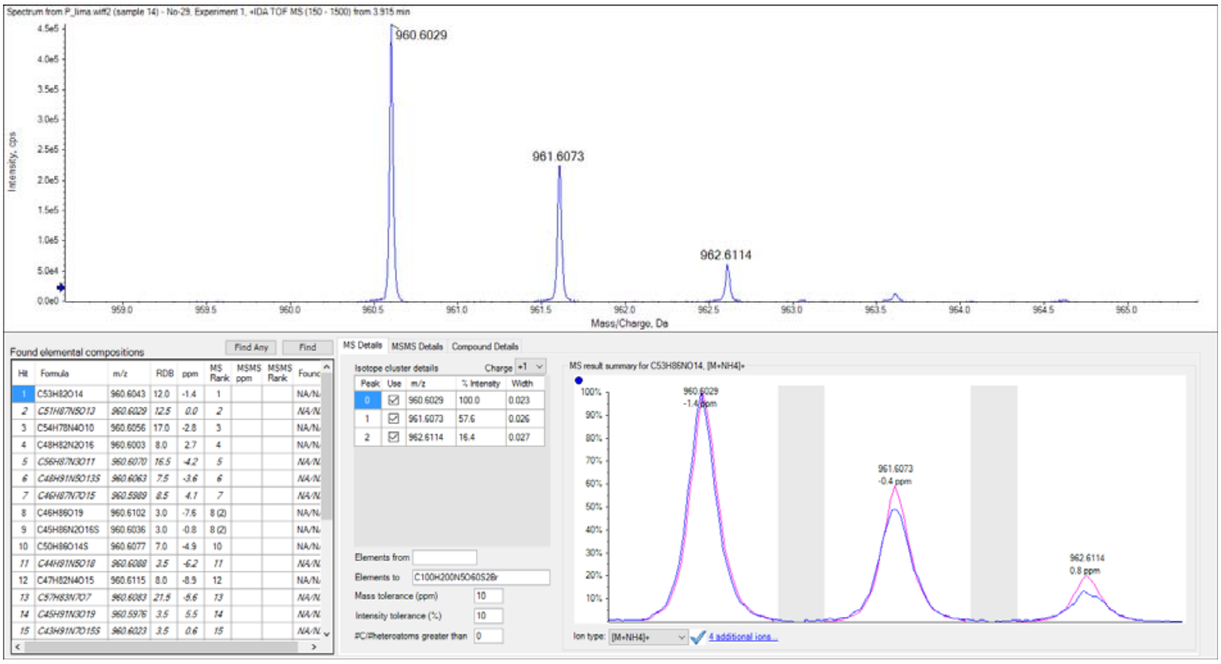

Figure S13. HRESIMS data of compound **3**.

(A)

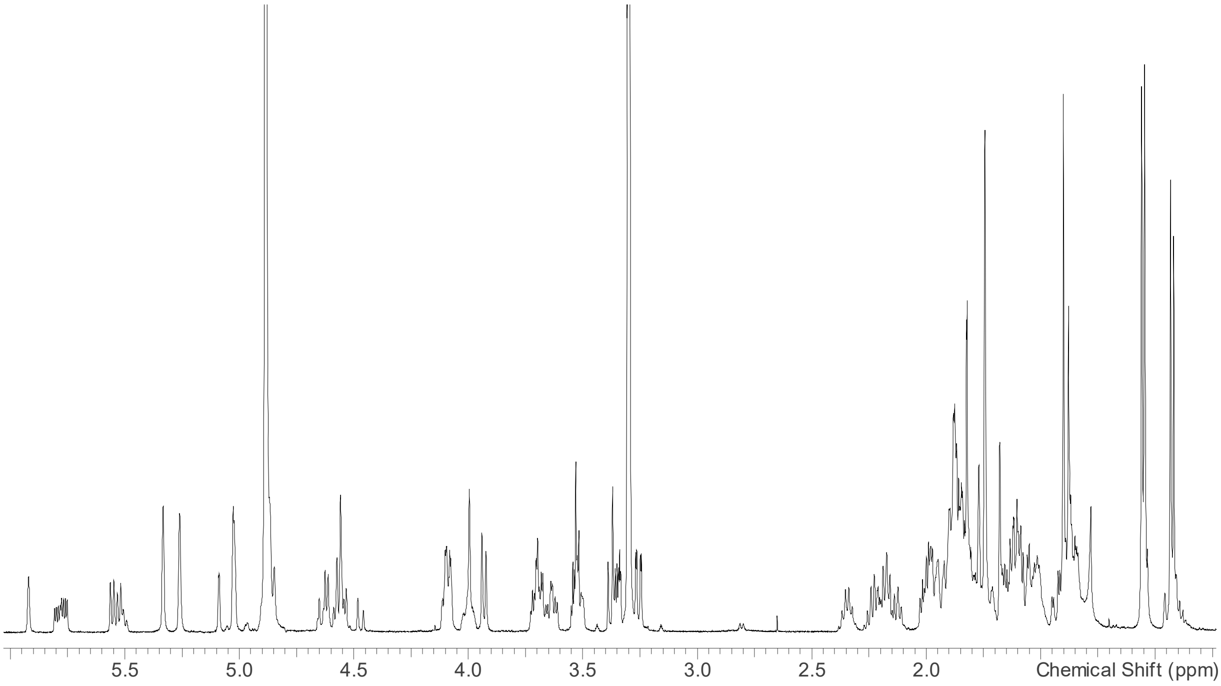

(B)

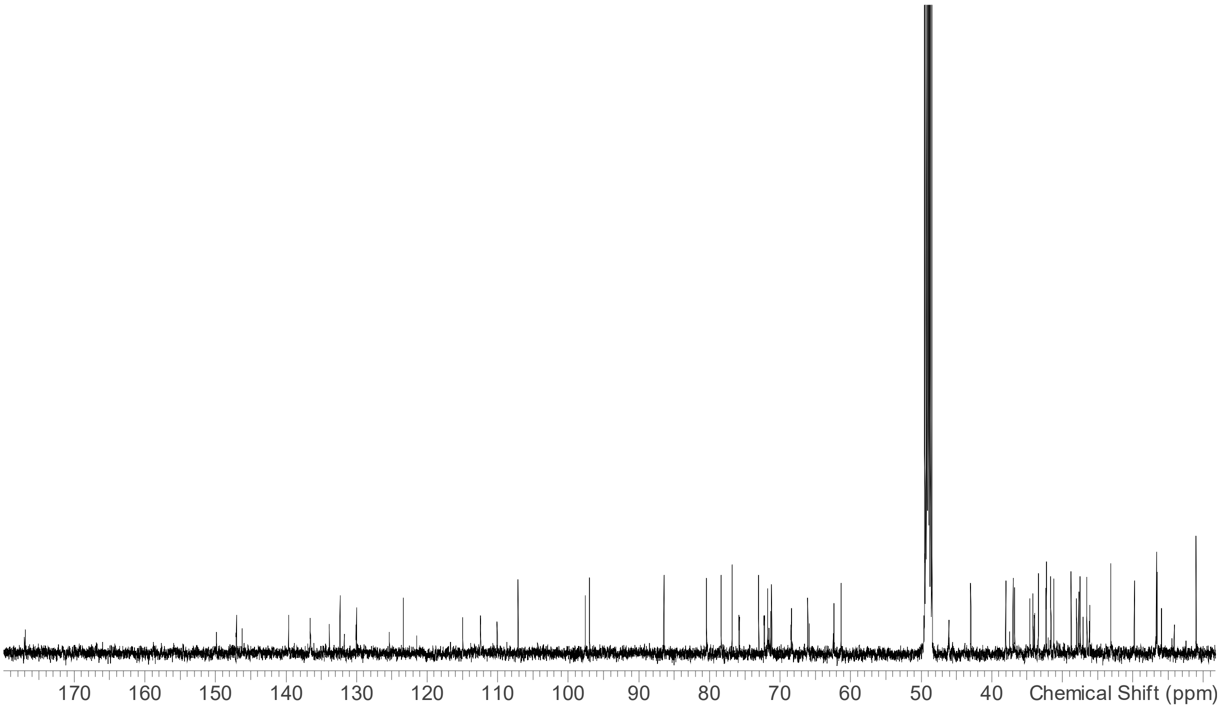

Figure S14. (A) ^{1}H NMR and (B)^{13}C NMR spectra of compound **3** measured at 500 MHz(^{1}H).

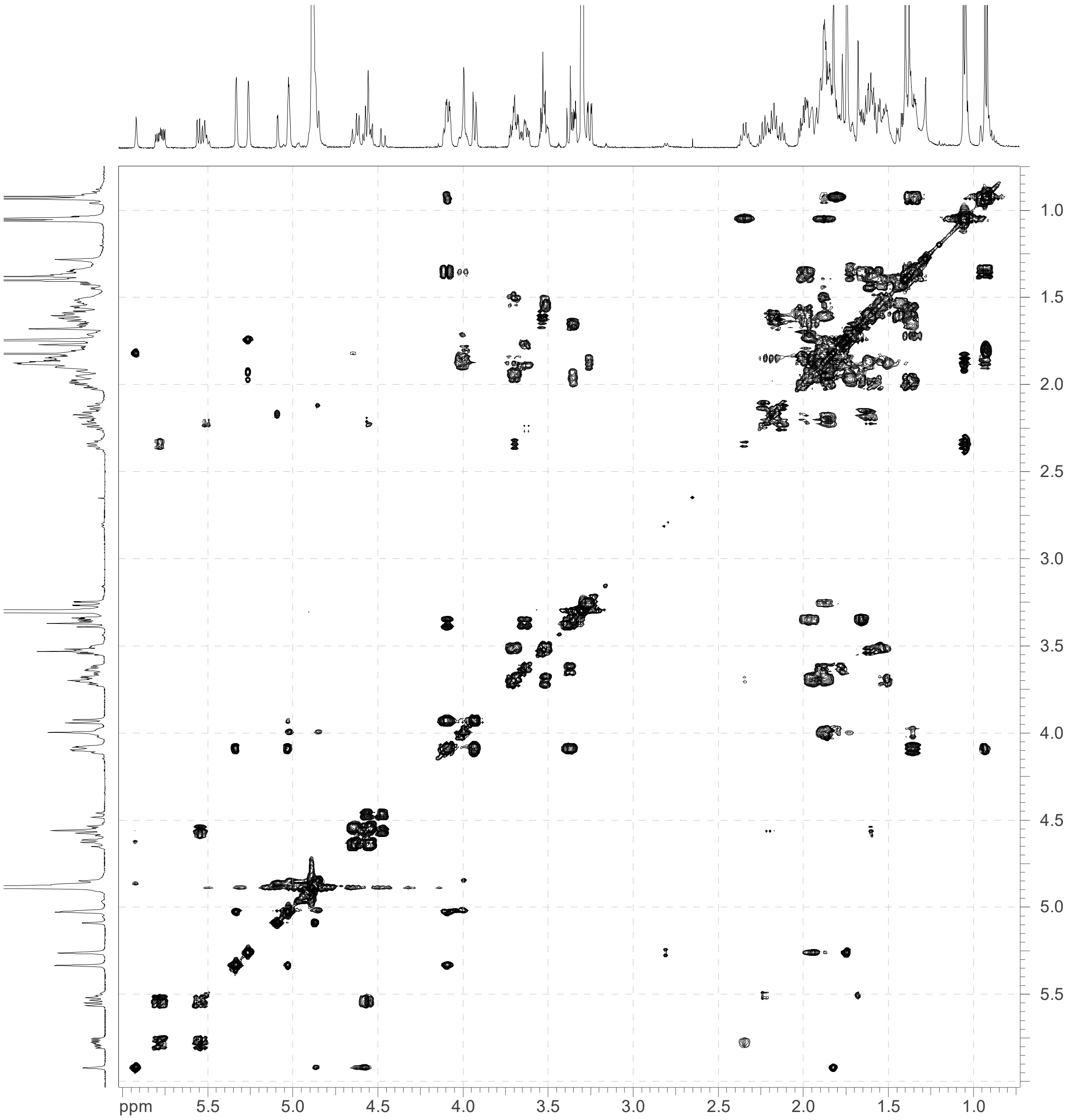

Figure S15. COSY spectrum of compound **3** measured at 500 MHz(^{1}H).

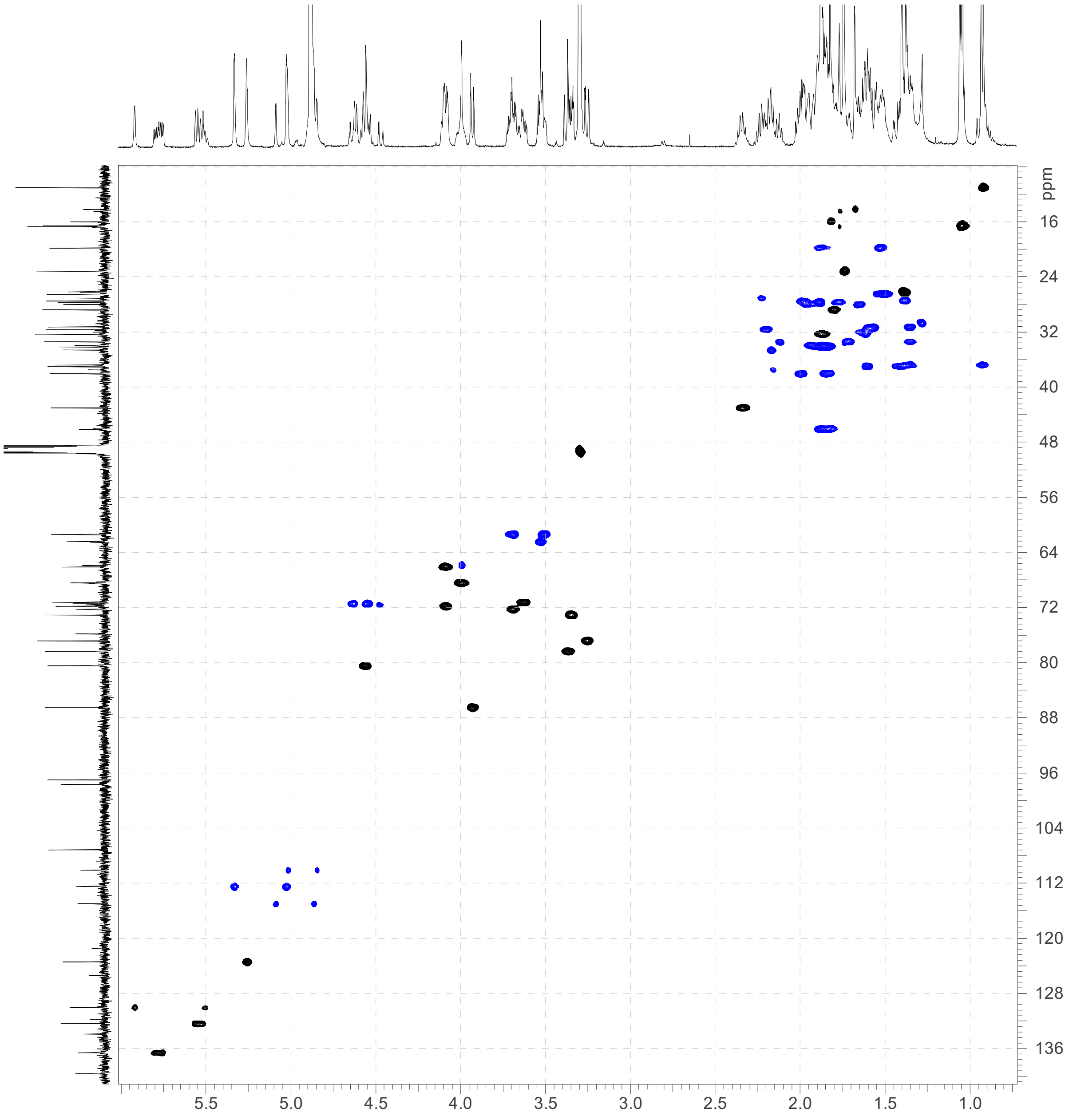

Figure S16. HSQC spectrum of compound **3** measured at 500 MHz(^{1}H).

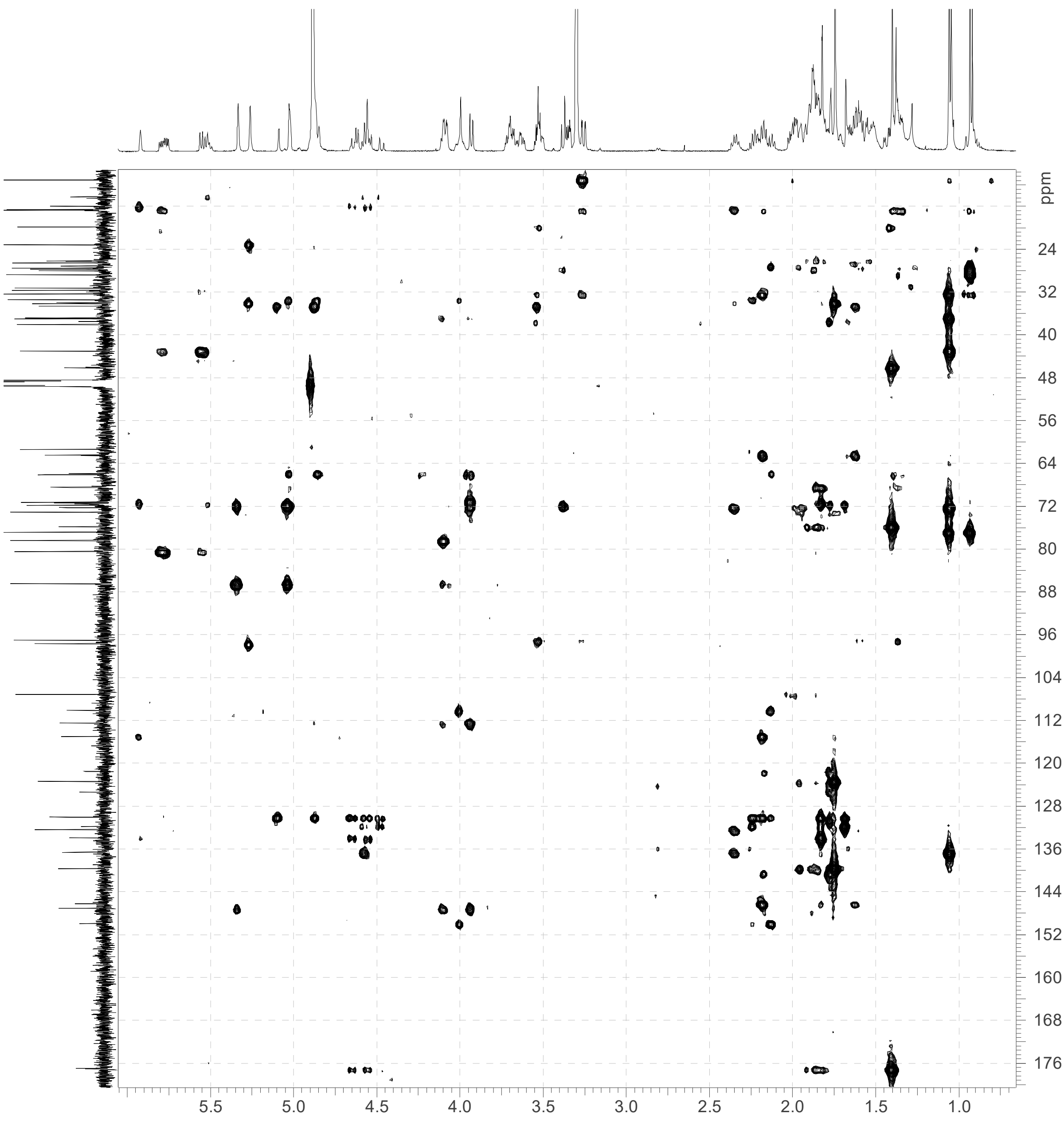

Figure S17. HMBC spectrum of compound **3** measured at 500 MHz(^{1}H).

Functional	Solvent?	Basis Set	Type of Data
mPW1PW91	PCM	6-311G(d,p)	Shielding Tensors

	Isomer 1	Isomer 2	Isomer 3	Isomer 4	Isomer 5	Isomer 6
sDP4+ (H data)	5.72%	94.28%	-	-	-	-
sDP4+ (C data)	99.83%	0.17%	-	-	-	-
sDP4+ (all data)	97.27%	2.73%	-	-	-	-
uDP4+ (H data)	43.89%	56.11%	-	-	-	-
uDP4+ (C data)	99.15%	0.85%	-	-	-	-
uDP4+ (all data)	98.92%	1.08%	-	-	-	-
DP4+ (H data)	4.53%	95.47%	-	-	-	-
DP4+ (C data)	100.00%	0.00%	-	-	-	-
DP4+ (all data)	99.97%	0.03%	-	-	-	-

Isomer 1 = (*E*) form diol

Isomer 2 = (*Z*) form diol

Figure S18. DP4+ probability analysis for the diol moiety of compound **3**.

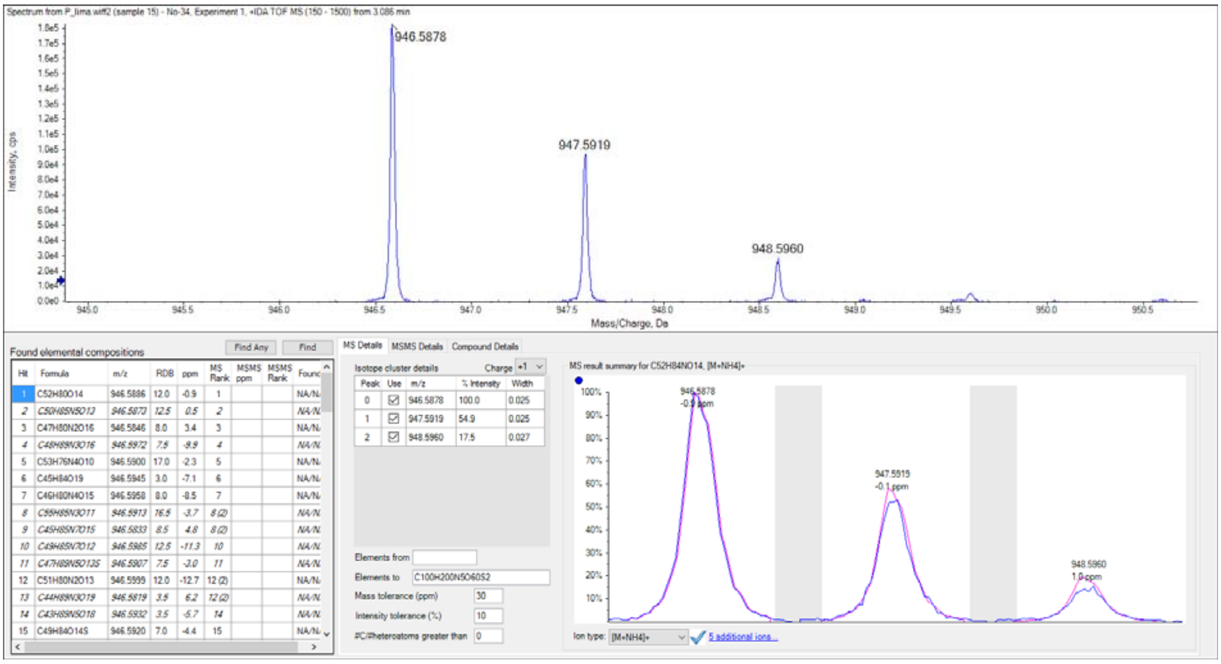

Figure S19. HRESIMS data of compound **4**.

(A)

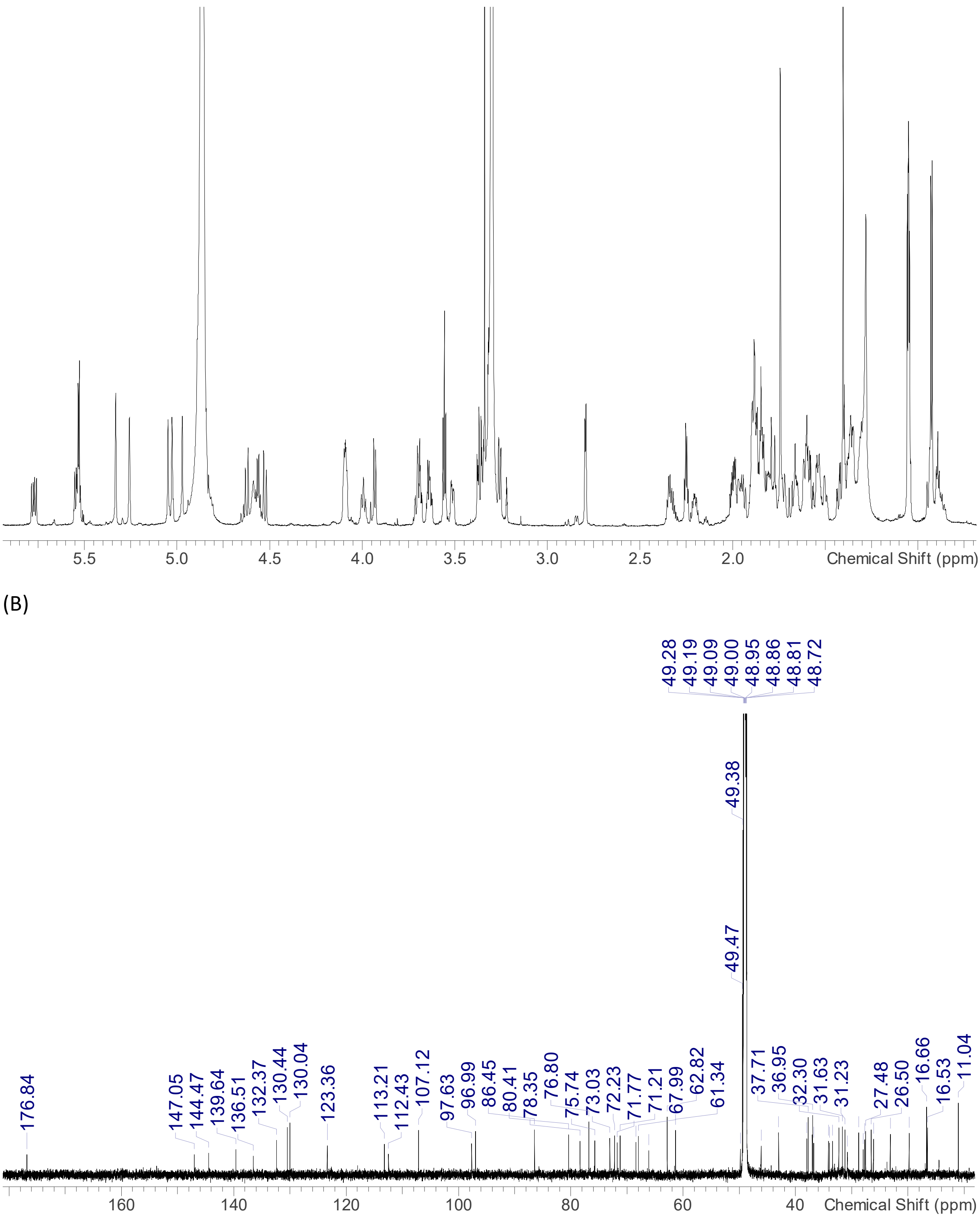

Figure S20. (A) ^{1}H NMR and (B)^{13}C NMR spectra of compound **4** measured at 900 MHz(^{1}H).

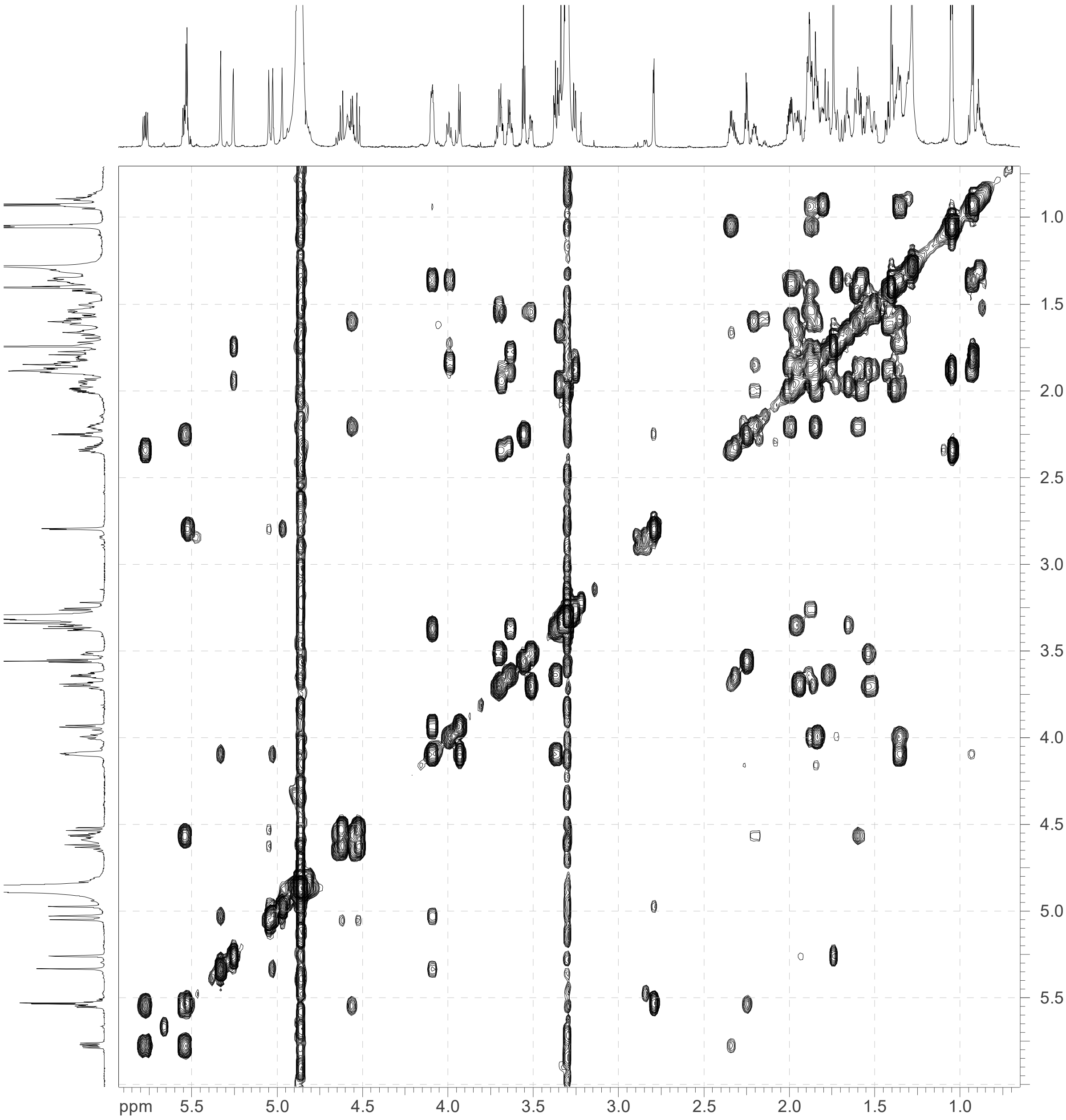

Figure S21. COSY spectrum of compound **4** measured at 900 MHz(^{1}H).

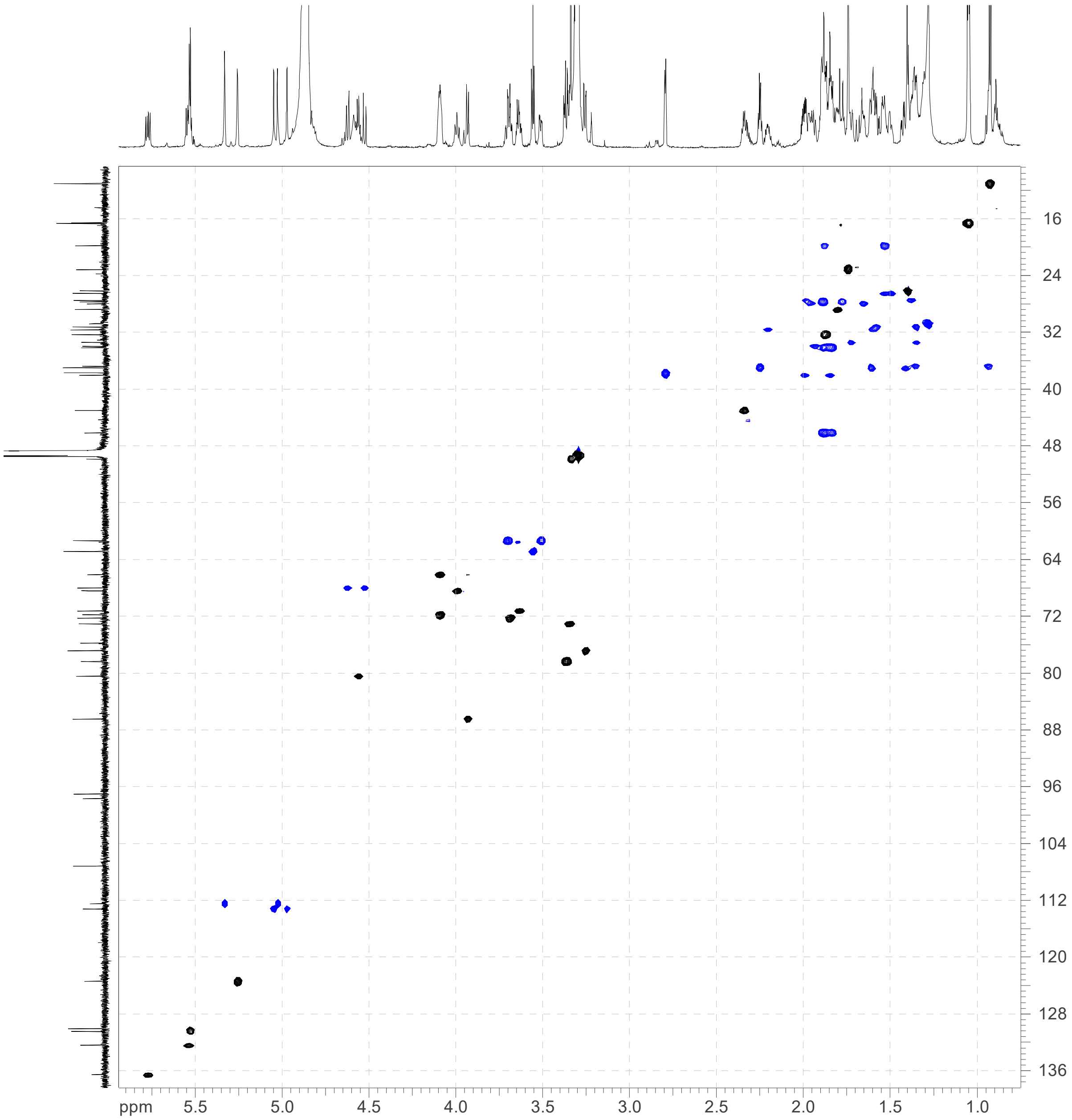

Figure S22. HSQC spectrum of compound **4** measured at 900 MHz(^{1}H).

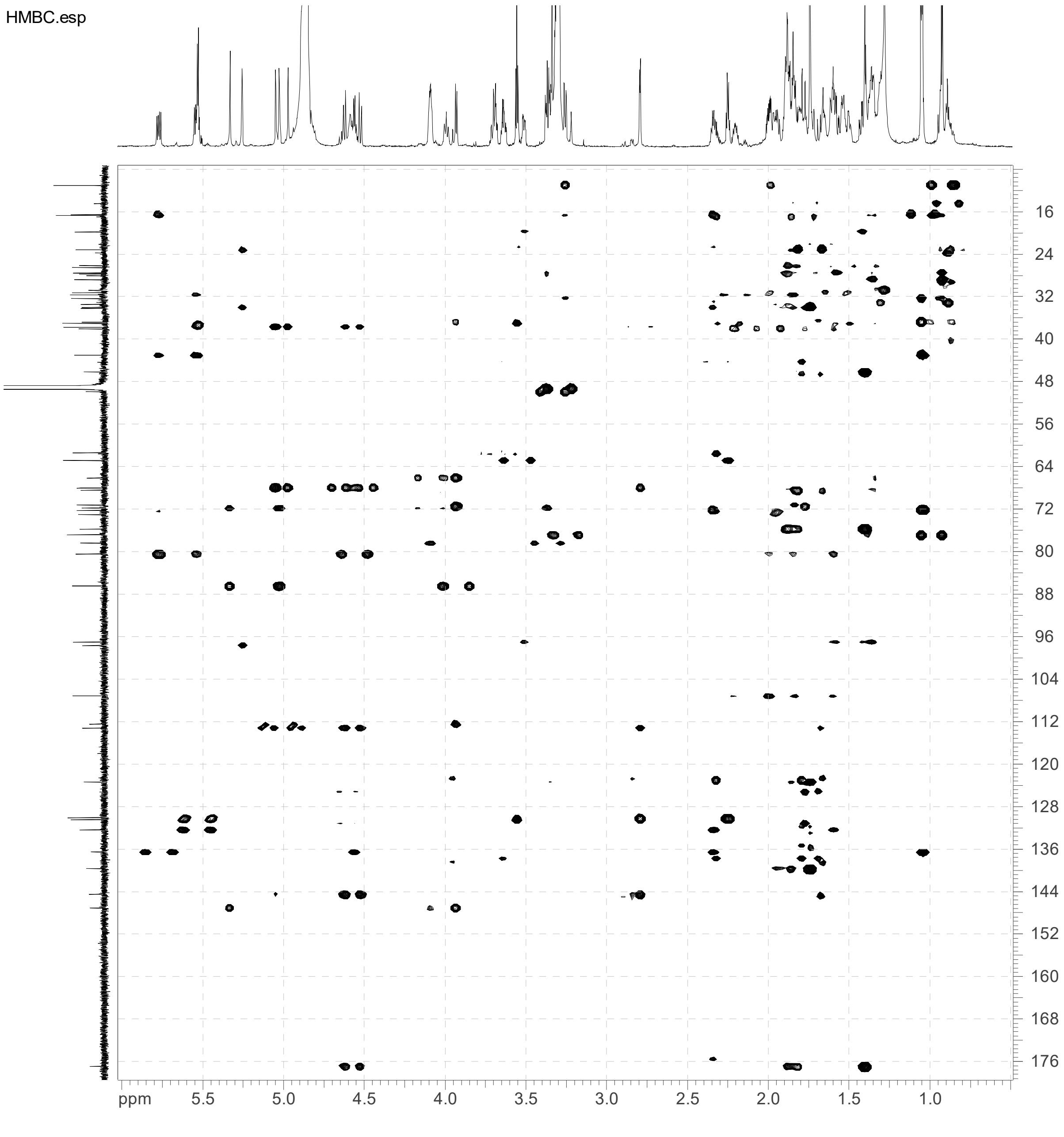

Figure S23. HMBC spectrum of compound **4** measured at 900 MHz(^{1}H).

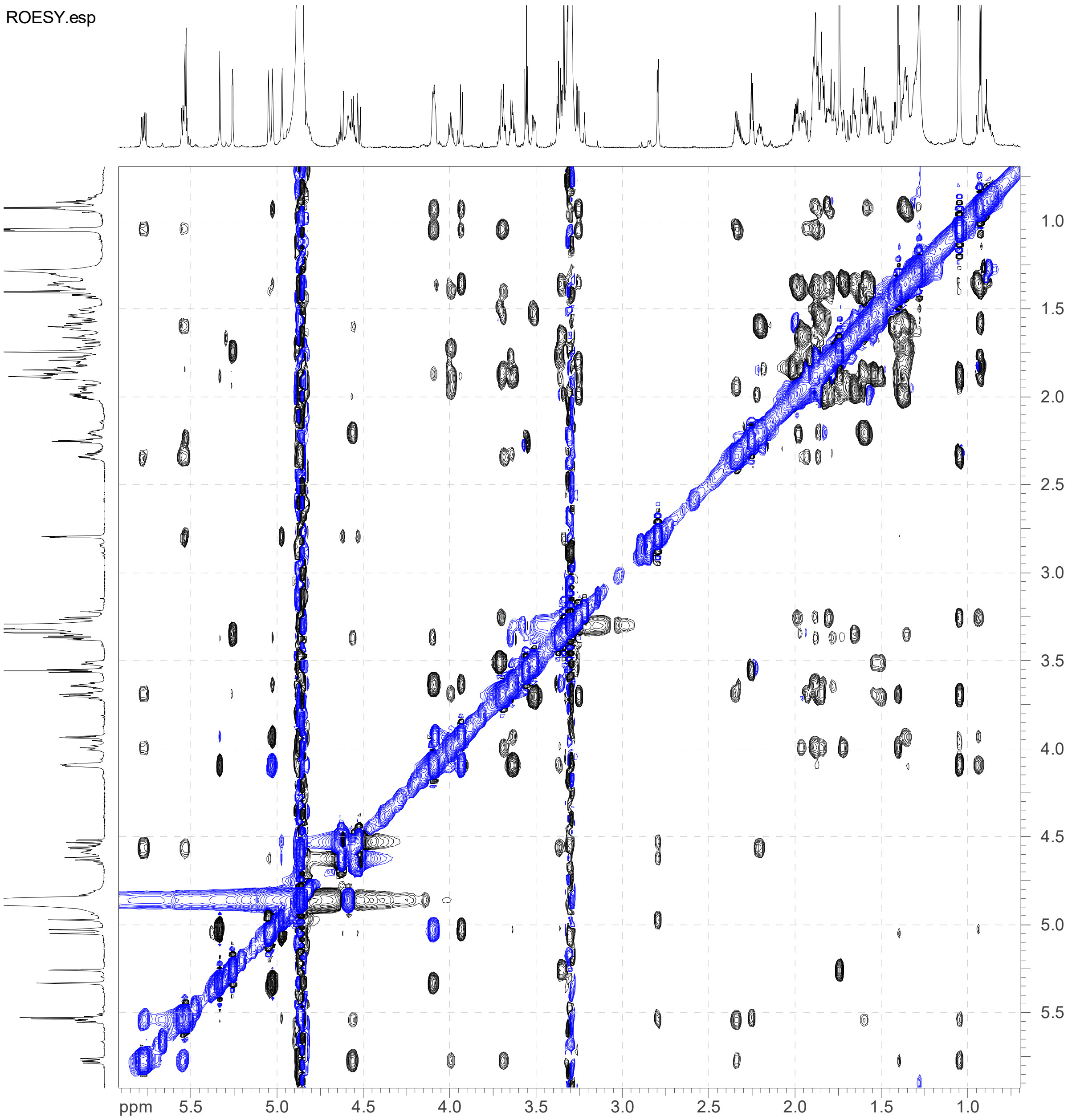

Figure S24. ROESY spectrum of compound **4** measured at 900 MHz(^{1}H).

(A)

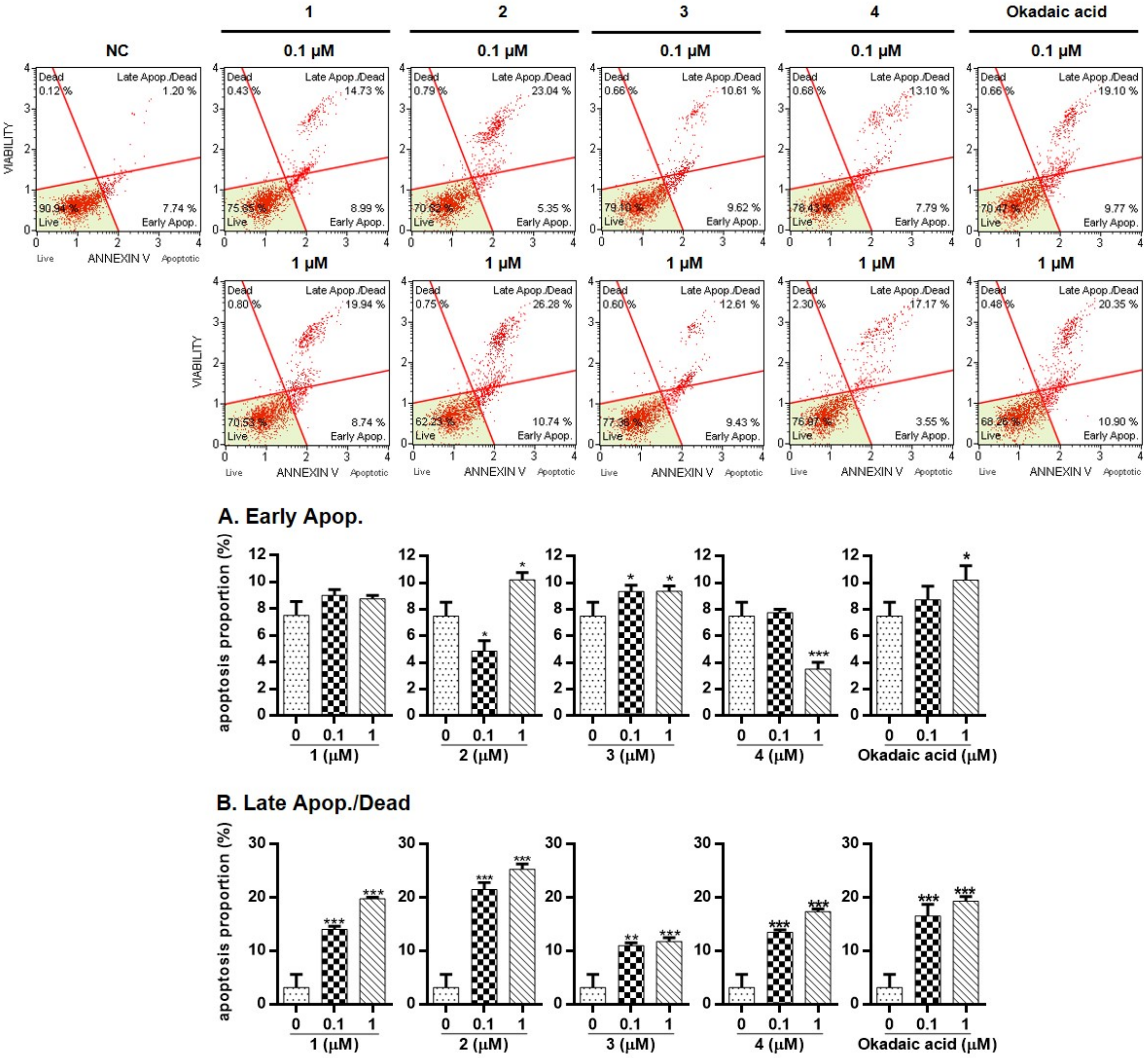
1
2
3
4
Okadaic acid
NC
0.1 μM
1 μM
Dead
Late Apop./Dead
Live
Early Apop.
VIABILITY
ANNEXIN V
Apoptotic
NC: 0.12 % 1.20 % 90.94 % 7.74 %
1, 0.1 μM: 0.43 % 14.73 % 75.85 % 8.99 %
2, 0.1 μM: 0.79 % 23.04 % 70.82 % 5.35 %
3, 0.1 μM: 0.66 % 10.61 % 79.10 % 9.62 %
4, 0.1 μM: 0.68 % 13.10 % 78.43 % 7.79 %
Okadaic acid, 0.1 μM: 0.66 % 19.10 % 70.47 % 9.77 %
1, 1 μM: 0.80 % 19.94 % 70.53 % 8.74 %
2, 1 μM: 0.75 % 26.28 % 62.23 % 10.74 %
3, 1 μM: 0.60 % 12.61 % 77.36 % 9.43 %
4, 1 μM: 2.30 % 17.17 % 76.97 % 3.55 %
Okadaic acid, 1 μM: 0.48 % 20.35 % 68.26 % 10.90 %
A. Early Apop.
B. Late Apop./Dead
apoptosis proportion (%)
0 0.1 1
1 (μM)
2 (μM)
3 (μM)
4 (μM)
Okadaic acid (μM)

(B)

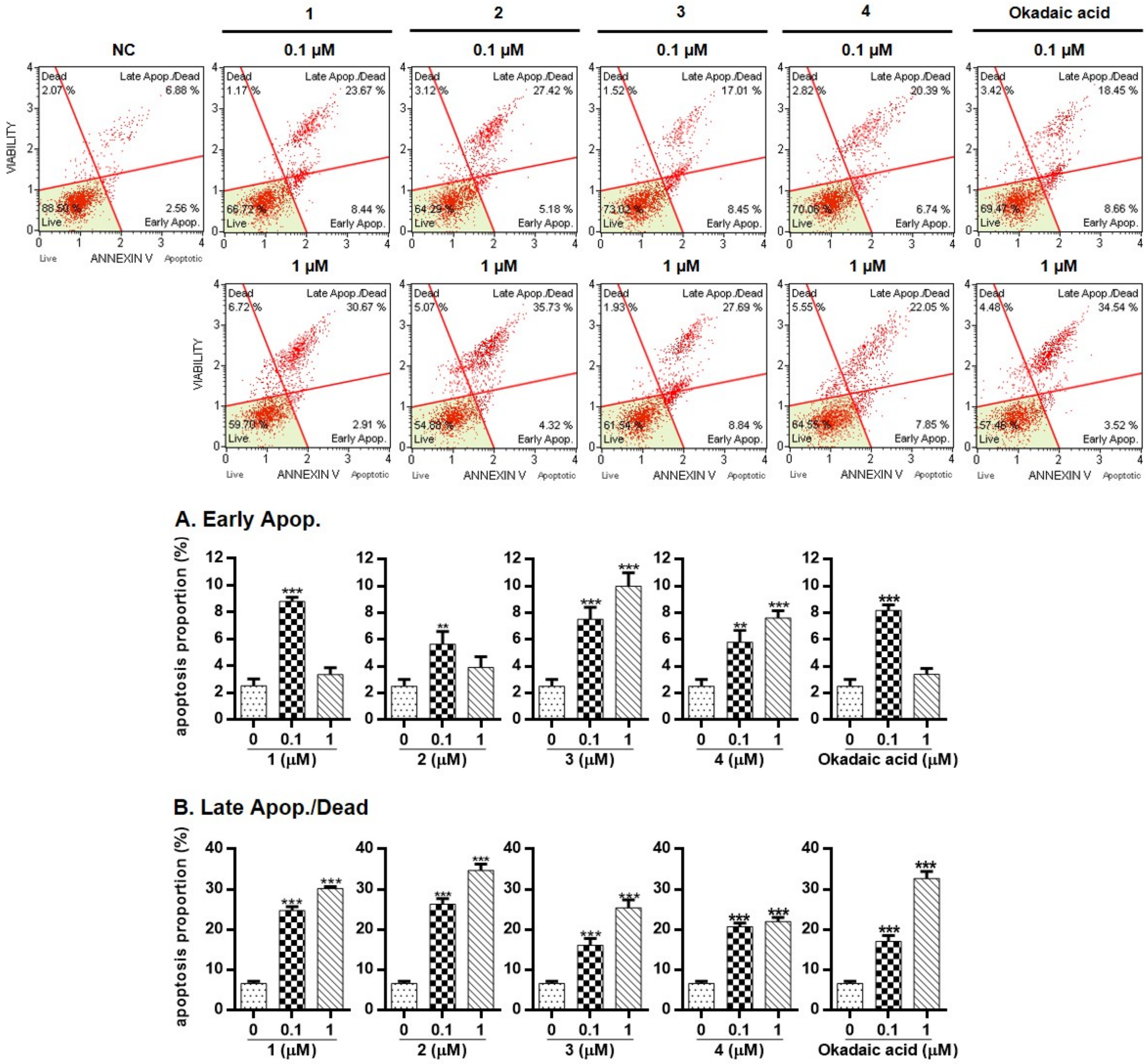

(C)

1 **2** **3** **4** **Okadaic acid**

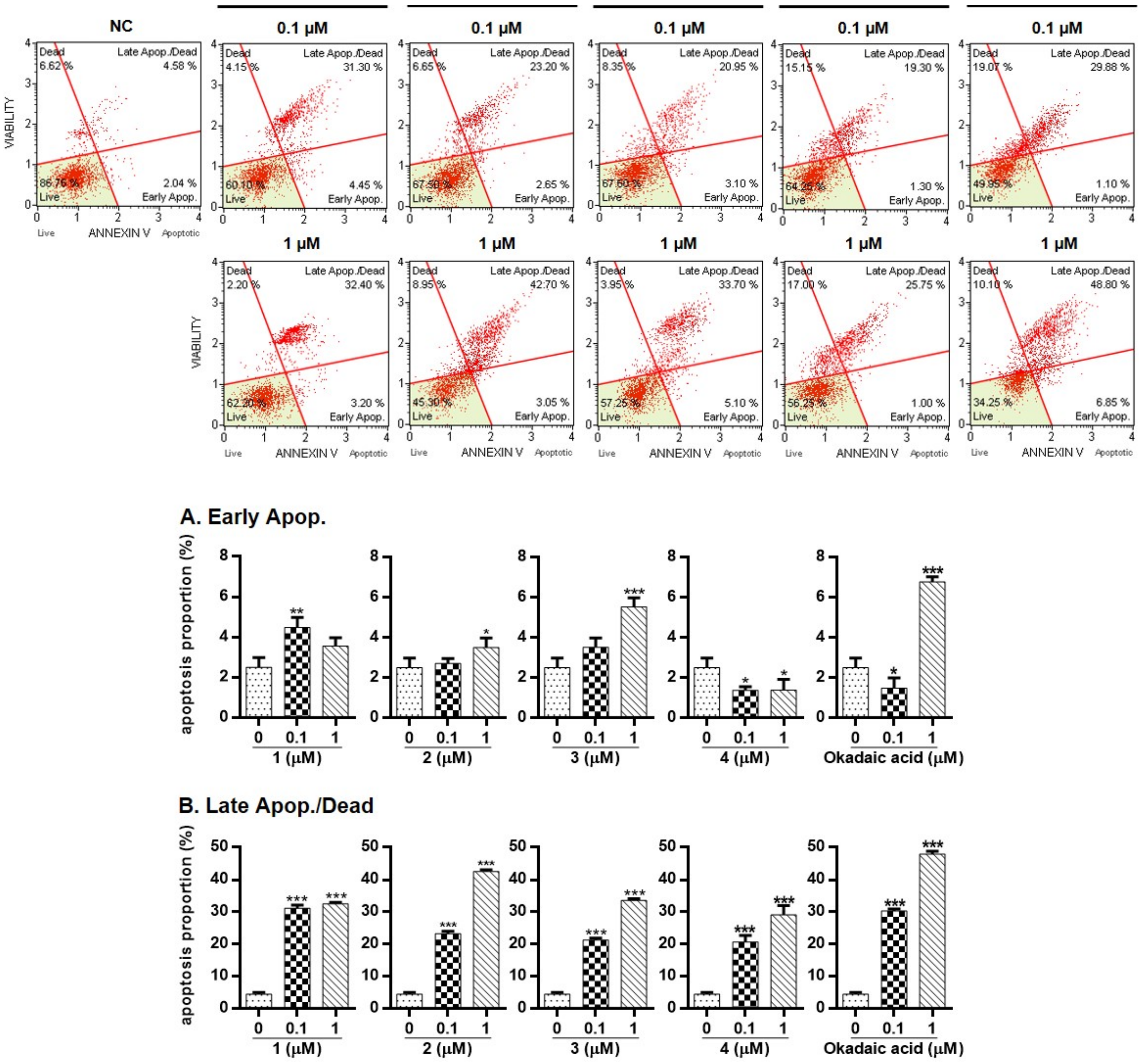

Figure S25. Apoptosis of (A) Neuro2a, (B) HCT116, and (C) HepG2 cells in the presence of compounds **1**–**4** or okadaic acid as determined using the Annexin-V/PI double staining assay.
